# Supplementary material for: Household structure, composition and child mortality in the unfolding antiretroviral therapy era in rural South Africa: comparative evidence from population surveillance, 2000–2015
Source: BMJ Open. 2023 Mar 15;13(3):e070388. doi: 10.1136/bmjopen-2022-070388 (PMC10030929; doi:10.1136/bmjopen-2022-070388)
Supplement: Supplementary data [file bmjopen-2022-070388supp001.pdf]

Supplemental Figure 1. Child mortality rates by year, Agincourt Health and Demographic Surveillance System (AHDSS) and Africa Health Research Institute (AHRI), South Africa 2000-2015.

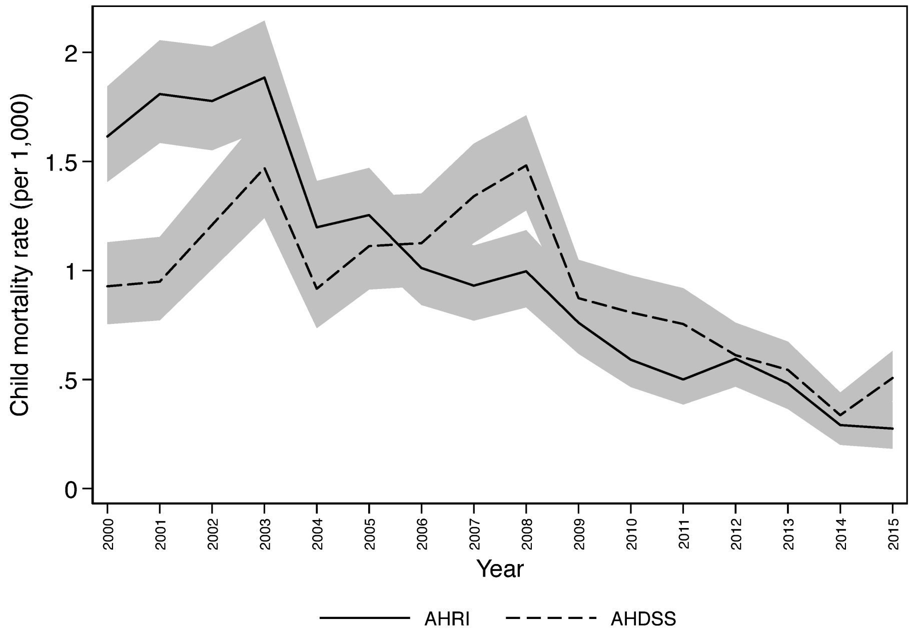

Supplemental Figure 2. Distribution of child causes of death over time, Agincourt Health and Demographic Surveillance System (AHDSS) and Africa Health Research Institute (AHRI), South Africa 2000-2007 and 2008-2015. Child causes of death classified by InterVA-5 based on VA.

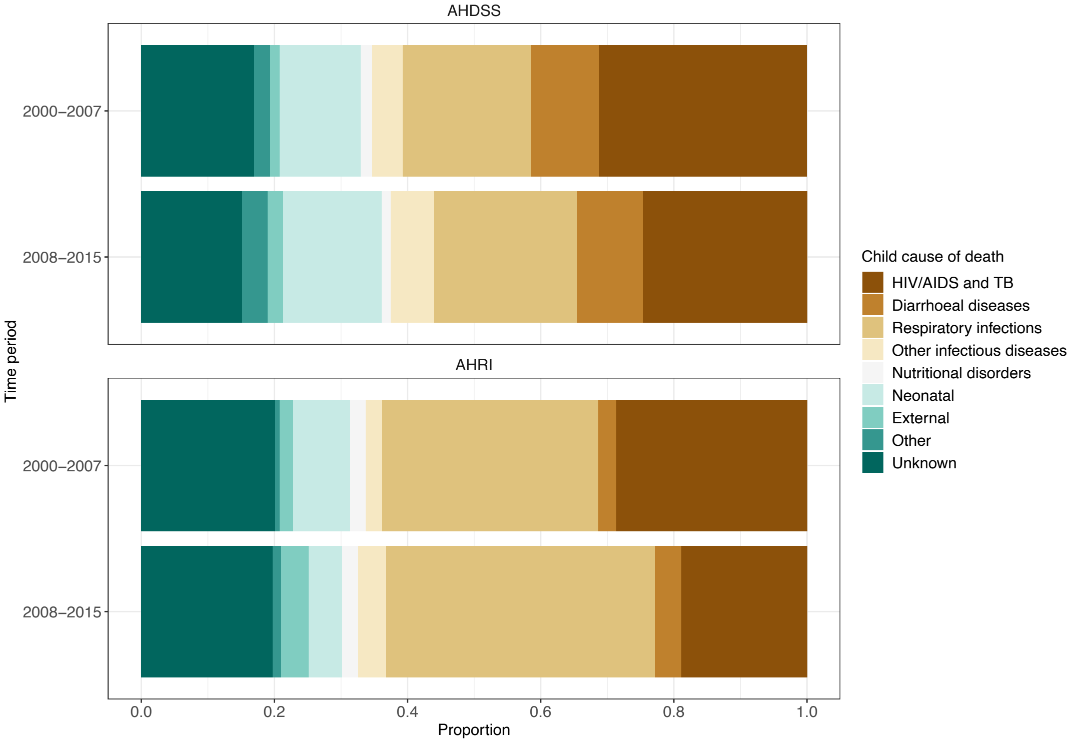

Supplemental Table 1. Multilevel relative risk regression of child death on household structure and controls, Agincourt Health and Demographic Surveillance System (AHDSS) and Africa Health Research Institute (AHRI), South Africa 2000-2015 (n=3,637,638 child months).

	RRR	95% CI	p-value
Site			
AHRI	1	-	
AHDSS	0.757	[0.647, 0.887]	0.001
Time period			
2000 to 2007	1	-	
2008 to 2015	0.415	[0.346, 0.498]	<0.001
Site X time period			
AHDSS X 2008 to 2015	1.454	[1.251, 1.689]	<0.001
Sex of child			
Female	1	-	
Male	1.124	[1.049, 1.204]	0.001
Child age (months)			
<1	1	-	
1 to 6	0.38	[0.342, 0.423]	<0.001
7 to 23	0.177	[0.159, 0.196]	<0.001
24 to 59	0.035	[0.031, 0.039]	<0.001
Multiple birth			
Singleton	1	-	
Multiple birth	1.818	[1.557, 2.124]	<0.001
Mother's age at birth (years			
15-19	0.864	[0.780, 0.956]	0.005
20-24	1	-	
25-29	1.264	[1.148, 1.392]	<0.001
30-34	1.089	[0.969, 1.225]	0.152
35+	1.237	[1.091, 1.403]	0.001
Number ages 0-5			
0	1	-	
1	1.098	[1.011, 1.192]	0.027
2+	1.251	[1.138, 1.376]	<0.001
Number ages 5-19			
0	1	-	
1 to 2	0.98	[0.849, 1.132]	0.787
3 to 4	0.928	[0.800, 1.076]	0.321
5+	1.044	[0.894, 1.219]	0.588
Number ages 20+			
0 to 2	1	-	
3 to 4	0.975	[0.879, 1.081]	0.631
5+	0.938	[0.841, 1.046]	0.249

Household structure			
Nuclear	1	-	
Vertical only	1.644	[1.325, 2.041]	<0.001
Horizontal/vertical	1.635	[1.408, 1.898]	<0.001
No parents, kin present	2.906	[2.169, 3.895]	<0.001
Mother only	1.554	[1.313, 1.839]	<0.001
Other	1.809	[1.411, 2.319]	<0.001
Site X household structure			
AHDSS X Vertical only	1.181	[0.892, 1.563]	0.245
AHDSS X Horizontal/vertical	1.07	[0.877, 1.305]	0.506
AHDSS X No parents, kin present	0.938	[0.595, 1.479]	0.784
AHDSS X Mother only	1.373	[1.112, 1.696]	0.003
AHDSS X Other	1.932	[1.339, 2.787]	<0.001
Time period X household structure			
2008 to 2015 X Vertical only	0.842	[0.632, 1.122]	0.241
2008 to 2015 X Horizontal/vertical	0.905	[0.742, 1.104]	0.324
2008 to 2015 X No parents, kin present	0.818	[0.506, 1.323]	0.413
2008 to 2015 X Mother only	1.2	[0.973, 1.479]	0.089
2008 to 2015 X Other	1.07	[0.725, 1.578]	0.734
	Parameter		
σ^2_{mother}	3.152	[2.574, 3.859]	

Supplemental Table 2. Multilevel relative risk regression of child death on kin presence and controls, Agincourt Health and Demographic Surveillance System (AHDSS) and Africa Health Research Institute (AHRI), South Africa 2000-2015 (n=3,637,638 child months).

	**RRR**	**95% CI**	**p-value**
Site			
AHRI	1	-	
AHDSS	0.874	[0.794, 0.961]	0.006
Time period			
2000 to 2007	1	-	
2008 to 2015	0.456	[0.399, 0.520]	<0.001
Site X time period			
AHDSS X 2008 to 2015	1.437	[1.241, 1.664]	<0.001
Sex of child			
Female	1	-	
Male	1.125	[1.050, 1.205]	0.001
Child age (months)			
<1	1	-	
1 to 6	0.379	[0.341, 0.422]	<0.001
7 to 23	0.176	[0.159, 0.195]	<0.001
24 to 59	0.035	[0.031, 0.039]	<0.001
Multiple birth			
Singleton	1	-	
Multiple birth	1.804	[1.545, 2.106]	<0.001
Mother's age at birth (years			
15-19	0.866	[0.780, 0.961]	0.007
20-24	1	-	
25-29	1.277	[1.155, 1.413]	<0.001
30-34	1.127	[0.995, 1.278]	0.06
35+	1.309	[1.143, 1.499]	<0.001
Number ages 0-5			
0	1	-	
1	1.096	[1.009, 1.190]	0.029
2+	1.249	[1.136, 1.373]	<0.001
Number ages 5-19			
0	1	-	
1 to 2	1.069	[0.917, 1.246]	0.395
3 to 4	1.014	[0.864, 1.189]	0.865
5+	1.147	[0.969, 1.357]	0.11
Number ages 20+			
0 to 2	1	-	
3 to 4	0.953	[0.861, 1.056]	0.359
5+	0.903	[0.811, 1.004]	0.06
Parental presence			

Both parents	1	-	
One parent	1.569	[1.363, 1.807]	<0.001
Neither parent	2.623	[2.058, 3.343]	<0.001
Related adult presence			
No	1	-	
Yes	2.954	[1.769, 4.931]	<0.001
Parental presence X related adult presence			
One parent X related adult present	0.316	[0.188, 0.530]	<0.001
Neither parent X related adult present	0.307	[0.171, 0.551]	<0.001
Related siblings			
No	1	-	
Yes	0.712	[0.606, 0.837]	<0.001
Parental presence X related siblings			
One parent X related siblings present	1.464	[1.233, 1.737]	<0.001
Neither parent X related siblings present	1.518	[1.070, 2.153]	0.019
Time period X related adult presence			
2008 to 2015 X related adult presence	0.806	[0.698, 0.932]	0.004
	Parameter		
σ^2_{mother}	1.128	[0.944, 1.349]	

Supplemental Table 3. Multilevel relative risk regression of child death on household structure and controls, Agincourt Health and Demographic Surveillance System (AHDSS) and Africa Health Research Institute (AHRI), South Africa 2000-2015 (n=3,637,638 child months). Estimation sample restricted to those with household SES information.

	**RRR**	**95% CI**	**p-value**
Site			
AHRI	1	-	
AHDSS	0.772	[0.625, 0.954]	0.017
Time period			
2000 to 2007	1	-	
2008 to 2015	0.391	[0.305, 0.501]	<0.001
Site X time period			
AHDSS X 2008 to 2015	1.395	[1.141, 1.705]	0.001
Sex of child			
Female	1	-	
Male	1.134	[1.037, 1.240]	0.006
Child age (months)			
<1	1	-	
1 to 6	0.355	[0.304, 0.415]	<0.001
7 to 23	0.176	[0.152, 0.205]	<0.001
24 to 59	0.036	[0.030, 0.042]	<0.001
Multiple birth			
Singleton	1	-	
Multiple birth	1.58	[1.281, 1.948]	<0.001
Mother's age at birth (years			
15-19	0.897	[0.785, 1.024]	0.107
20-24	1	-	
25-29	1.364	[1.202, 1.548]	<0.001
30-34	1.175	[1.008, 1.368]	0.039
35+	1.391	[1.183, 1.634]	<0.001
Number ages 0-5			
0	1	-	
1	1.049	[0.943, 1.167]	0.375
2+	1.225	[1.082, 1.387]	0.001
Number ages 5-19			
0	1	-	
1 to 2	1.031	[0.855, 1.243]	0.746
3 to 4	0.949	[0.782, 1.152]	0.594
5+	1.034	[0.843, 1.268]	0.748
Number ages 20+			
0 to 2	1	-	
3 to 4	0.912	[0.798, 1.041]	0.174
5+	0.868	[0.753, 1.002]	0.053

Household structure			
Nuclear	1	-	
Vertical only	1.851	[1.374, 2.495]	<0.001
Horizontal/vertical	1.733	[1.408, 2.131]	<0.001
No parents, kin present	3.356	[2.292, 4.914]	<0.001
Mother only	1.716	[1.357, 2.170]	<0.001
Other	1.892	[1.335, 2.680]	<0.001
Site X household structure			
AHDSS X Vertical only	1.099	[0.752, 1.604]	0.627
AHDSS X Horizontal/vertical	1.127	[0.863, 1.470]	0.38
AHDSS X No parents, kin present	1.072	[0.619, 1.857]	0.804
AHDSS X Mother only	1.309	[0.981, 1.746]	0.067
AHDSS X Other	2.069	[1.274, 3.360]	0.003
Time period X household structure			
2008 to 2015 X Vertical only	0.734	[0.502, 1.075]	0.112
2008 to 2015 X Horizontal/vertical	0.838	[0.648, 1.085]	0.181
2008 to 2015 X No parents, kin present	0.763	[0.416, 1.399]	0.382
2008 to 2015 X Mother only	1.278	[0.978, 1.669]	0.073
2008 to 2015 X Other	1.044	[0.624, 1.745]	0.87
Household SES			
Low	1	-	
Middle	0.949	[0.852, 1.058]	0.346
High	0.788	[0.702, 0.883]	<0.001
Household head gender			
Female	1	-	
Male	1.104	[0.999, 1.220]	0.052
	Parameter		
σ^2_{mother}	3.152	[2.574, 3.859]	

Supplemental Table 4. Multilevel relative risk regression of child death on kin presence and controls, Agincourt Health and Demographic Surveillance System (AHDSS) and Africa Health Research Institute (AHRI), South Africa 2000-2015 (n=2,468,466 child months). Estimation sample restricted to those with household SES information.

	**RRR**	**95% CI**	**p-value**
Site			
AHRI	1	-	
AHDSS	0.903	[0.797, 1.022]	0.106
Time period			
2000 to 2007	1	-	
2008 to 2015	0.442	[0.367, 0.532]	<0.001
Site X time period			
AHDSS X 2008 to 2015	1.374	[1.129, 1.672]	0.002
Sex of child			
Female	1	-	
Male	1.136	[1.039, 1.242]	0.005
Child age (months)			
<1	1	-	
1 to 6	0.354	[0.303, 0.414]	<0.001
7 to 23	0.176	[0.152, 0.204]	<0.001
24 to 59	0.036	[0.030, 0.042]	<0.001
Multiple birth			
Singleton	1	-	
Multiple birth	1.571	[1.275, 1.936]	<0.001
Mother's age at birth (years			
15-19	0.889	[0.775, 1.019]	0.091
20-24	1	-	
25-29	1.4	[1.227, 1.597]	<0.001
30-34	1.246	[1.059, 1.466]	0.008
35+	1.523	[1.280, 1.812]	<0.001
Number ages 0-5			
0	1	-	
1	1.046	[0.941, 1.163]	0.406
2+	1.219	[1.077, 1.380]	0.002
Number ages 5-19			
0	1	-	
1 to 2	1.19	[0.974, 1.454]	0.089
3 to 4	1.103	[0.894, 1.360]	0.36
5+	1.212	[0.971, 1.513]	0.089
Number ages 20+			
0 to 2	1	-	
3 to 4	0.89	[0.780, 1.015]	0.081
5+	0.827	[0.719, 0.951]	0.008

Parental presence			
Both parents	1	-	
One parent	1.609	[1.341, 1.930]	<0.001
Neither parent	2.865	[2.086, 3.933]	<0.001
Related adult presence			
No	1	-	
Yes	4.031	[2.137, 7.604]	<0.001
Parental presence X related adult presence			
One parent X related adult present	0.228	[0.120, 0.432]	<0.001
Neither parent X related adult present	0.229	[0.110, 0.474]	<0.001
Related siblings			
No	1	-	
Yes	0.6	[0.487, 0.739]	<0.001
Parental presence X related siblings			
One parent X related siblings present	1.695	[1.360, 2.113]	<0.001
Neither parent X related siblings present	1.84	[1.185, 2.856]	0.007
Time period X related adult presence			
2008 to 2015 X related adult presence	0.722	[0.599, 0.871]	0.001
Household SES			
Low	1	-	
Middle	0.946	[0.849, 1.054]	0.314
High	0.784	[0.699, 0.879]	<0.001
Household head gender			
Female	1	-	
Male	1.107	[1.004, 1.221]	0.042
	Parameter		
σ^2_{mother}	3.375	[2.494, 4.568]	
