# Supplementary material for: Household structure, composition and child mortality in the unfolding antiretroviral therapy era in rural South Africa: comparative evidence from population surveillance, 2000–2015
Source: BMJ Open. 2023 Mar 15;13(3):e070388. doi: 10.1136/bmjopen-2022-070388 (PMC10030929; doi:10.1136/bmjopen-2022-070388)
Supplement: Author's manuscript [file bmjopen-2022-070388.draft_revisions.pdf]

BMJ Open is committed to open peer review. As part of this commitment we make the peer review history of every article we publish publicly available.

When an article is published we post the peer reviewers' comments and the authors' responses online. We also post the versions of the paper that were used during peer review. These are the versions that the peer review comments apply to.

The versions of the paper that follow are the versions that were submitted during the peer review process. They are not the versions of record or the final published versions. They should not be cited or distributed as the published version of this manuscript.

BMJ Open is an open access journal and the full, final, typeset and author-corrected version of record of the manuscript is available on our site with no access controls, subscription charges or pay-per-view fees (http://bmjopen.bmj.com).

If you have any questions on BMJ Open's open peer review process please email info.bmjopen@bmj.com

BMJ Open

Household structure, composition, and child mortality in the unfolding antiretroviral therapy era in rural South Africa: Comparative evidence from population surveillance, 2000-2015

Journal:	*BMJ Open*
Manuscript ID	bmjopen-2022-070388
Article Type:	Original research
Date Submitted by the Author:	21-Nov-2022

Complete List of Authors:	Houle , Brian ; Australian National University, School of Demography; Faculty of Health Sciences University of the Witwatersrand<br>Kabudula, Chodziwadziwa; University of the Witwatersrand School of Public Health, MRC/Wits Rural Public Health and Health Transitions Research Unit (Agincourt),<br>Gareta, Dickman; Africa Health Research Institute, Statistics and Data<br>Herbst, Kobus; Africa Health Research Institute; DSI-MRC South African Population Research Infrastructure Network (SAPRIN)<br>Clark, Samuel J; The Ohio State University, Department of Sociology; Faculty of Health Sciences University of the Witwatersrand

Keywords:	Public health < INFECTIOUS DISEASES, Epidemiology < TROPICAL MEDICINE, HIV & AIDS < INFECTIOUS DISEASES

SCHOLARONE™
Manuscripts

I, the Submitting Author has the right to grant and does grant on behalf of all authors of the Work (as defined in the below author licence), an exclusive licence and/or a non-exclusive licence for contributions from authors who are: i) UK Crown employees; ii) where BMJ has agreed a CC-BY licence shall apply, and/or iii) in accordance with the terms applicable for US Federal Government officers or employees acting as part of their official duties; on a worldwide, perpetual, irrevocable, royalty-free basis to BMJ Publishing Group Ltd ("BMJ") its licensees and where the relevant Journal is co-owned by BMJ to the co-owners of the Journal, to publish the Work in this journal and any other BMJ products and to exploit all rights, as set out in our licence.

The Submitting Author accepts and understands that any supply made under these terms is made by BMJ to the Submitting Author unless you are acting as an employee on behalf of your employer or a postgraduate student of an affiliated institution which is paying any applicable article publishing charge ("APC") for Open Access articles. Where the Submitting Author wishes to make the Work available on an Open Access basis (and intends to pay the relevant APC), the terms of reuse of such Open Access shall be governed by a Creative Commons licence – details of these licences and which Creative Commons licence will apply to this Work are set out in our licence referred to above.

Other than as permitted in any relevant BMJ Author's Self Archiving Policies, I confirm this Work has not been accepted for publication elsewhere, is not being considered for publication elsewhere and does not duplicate material already published. I confirm all authors consent to publication of this Work and authorise the granting of this licence.

For peer review only

Household structure, composition, and child mortality in the unfolding antiretroviral therapy era in rural South Africa: Comparative evidence from population surveillance, 2000-2015

Brian Houle[1,2,3*]

Chodziwadziwa W Kabudula[2]

Dickman Gareta[4]

Kobus Herbst[4,5]

Samuel J Clark[2,3,6]

[*] Corresponding author: 146 Ellery Crescent, Acton, ACT 0200. brian.houle@anu.edu.au. +61 6125 7355.

1 School of Demography, The Australian National University, Canberra, Australia

2 MRC/Wits Rural Public Health and Health Transitions Research Unit (Agincourt), School of Public Health, Faculty of Health Sciences, University of the Witwatersrand, Johannesburg, South Africa

3 CU Population Center, Institute of Behavioral Science, University of Colorado at Boulder, Boulder, Colorado, USA

4 Africa Health Research Institute, KwaZulu-Natal, South Africa

5 DSI-MRC South African Population Research Infrastructure Network (SAPRIN), Durban, South Africa

6 Department of Sociology, The Ohio State University, Columbus, Ohio, USA

Word count: 2,698

For peer review only

ABSTRACT

Objectives: The structure and composition of the household has important influences on child mortality. However, little is known about these factors in HIV-endemic areas and how associations may change with the introduction and widespread availability of antiretroviral treatment (ART). We use comparative, longitudinal data from two demographic surveillance sites in rural South Africa (2000-2015) on mortality of children younger than five years (n=101,105).

Design: We use multilevel discrete time event history analysis to estimate children's probability of dying by their matrilineal residential arrangements. We also test if associations have changed over time with ART availability.

Setting: Rural South Africa.

Participants: Children younger than five years (n=101,105).

Results: 3,603 children died between 2000-2015. Mortality risks differed by co-residence patterns along with different types of kin present in the household. Children in nuclear households with both parents had the lowest risk of dying compared to all other household types. Associations with kin and child mortality were moderated by parental status. Having older siblings lowered the probability of dying only for children in a household with both parents (relative risk ratio (RRR)=0.736 95% CI [0.633, 0.855]). Only in the later ART period was there evidence that older adult kin lowered the probability of dying for children in single parent households (RRR=0.753 95% CI [0.664, 0.853]).

Conclusions: Our findings provide comparative evidence of how differential household profiles may place children at higher mortality risk. Formative research is needed to understand the role of other household kin in promoting child well-being, particularly in one-parent households that are increasingly prevalent.

Keywords: child mortality; household; HIV/AIDS; South Africa; longitudinal

For peer review only

Strengths and limitations of this study

- We provide 16 years of prospective, comparative population data from two rural areas of South Africa heavily impacted by HIV/AIDS on associations between household structure and composition with child mortality.
- Our measure of household kin is based on household memberships, meaning we cannot account for the role of kin who are members of another household.
- Our study was only able to identify the presence of matrilineal members in the household given limitations with father linkages over time.
- Formative research is needed to understand the role of other household kin in promoting child wellbeing, particularly in one-parent households.
- As HIV-endemic settings are continuing to undergo rapid demographic, epidemiological, and social transitions, further longitudinal research is needed to understand continued changes in living arrangements and the role of parents and kin in protecting the well-being of children.

For peer review only

INTRODUCTION

Reducing preventable child mortality remains an urgent global priority and central Sustainable Development Goal. Goal 3 targets reducing under-five mortality to 25 per 1,000 live births by 2030. While many countries are on track or have already reached this goal, the remaining global mortality burden is uneven. The majority of global under-five deaths occur in sub-Saharan Africa.[1] A greater understanding of the social, cultural, and contextual determinants of child mortality is critical to reduce child mortality in the region.

In the context of the HIV epidemic, a number of studies in sub-Saharan Africa have examined factors that impact child mortality, such as parental survival, [2–4] and HIV infection and antiretroviral therapy.[5–7] Household studies have focused on the role of older household members, fostering, and orphanhood.[8–12] Less attention has been paid to other contextual risk factors, especially with mortality changes associated with the HIV epidemic and more recently with the availability of antiretroviral therapy (ART). While there is a substantial literature from low and middle-income countries on the importance of factors such as household composition and child mortality,[13–18] there remains limited longitudinal evidence from HIV-endemic areas.[3, 19] The availability of antiretroviral therapy (ART) may also have changed the relative importance of different risk factors and how they influence child mortality[6] – highlighting the need for longitudinal data examining risk factors over time.

The extent to which studies are generalisable across settings is also unclear, particularly given differences in factors such as household organization and available resources. There is a lack of comparative research within countries, particularly amongst disadvantaged populations.[20] Understanding if and how household risk factors are associated with child mortality between different settings has important implications for policies and interventions to reduce preventable child mortality. Comparative evidence is needed to inform these efforts.

Our primary aim is to investigate the relationship between a child's risk of dying and their household's structure and composition. We follow the conceptual approach proposed by Madhavan et al.[21] by examining two household-level dimensions: (1) structure, referring to the configuration of generations in the household and level of nucleation; and (2) composition, referring to the availability of specific kin that may be moderated by parental

For peer review only

presence. We use comparative, longitudinal data from 2000-2015 from two demographic surveillance sites (DSS) in rural South Africa to examine these factors. The time period covers when ART was unavailable and after widespread availability, allowing for an assessment of if and how associations between different risk factors and child mortality have changed over time with ART availability.

METHOD

Setting

We used household census data from 2000-2015 for two populations living in rural South Africa. Both DSSs monitor a geographically defined population over time and collect prospective information, including demographic indicators (e.g., mortality, fertility, migration) as well as household-level information and social indicators. The first, the Agincourt Health and socio-Demographic Surveillance System (AHDSS), has been conducting an annual census update of the population since 1992, updating vital events (births, deaths, migration) along with socio-demographic information.[22] AHDSS is located in the Ehlanseni district of Mpumalanga. The primary ethnic group is amaShangaan. The population under surveillance was approximately 90,000 people in 2011. Until recently child and adult mortality were increasing.[23] ART became available in 2008, with resulting substantial reductions in mortality.[23]

The second site, the Africa Health Research Institute (AHRI), started bi-annual census updates in 2000.[24] AHRI is located in the Umkanyakude district of KwaZulu-Natal. The population is almost entirely Zulu-speaking and comprised approximately 90,000 people in 2011. HIV/AIDS has had a significant impact on the population, but the availability of ART in 2004 significantly increased life expectancy in the population.[25]

Household measures

Both DSSs update a roster of household members at each census update. To create a harmonized data structure across the sites, we used the household membership data (which included membership start and end dates) to identify members of the same household as the index child.

For peer review only

Our key measures were household structure and composition. We followed the categorisations of Madhavan et al.[21] as their analysis was based at AHDSS and included a complementary, cross-sectional data set with detailed kinship data. The structural typology included only those kin co-resident in the household: (1) both parents and no other kin (nuclear); (2) one or both parents and grandmother (vertical); (3) one or both parents, aunts/ uncles (horizontal); (4) one or both parents, grandmother and aunts/uncles (vertical and horizontal); (5) no parents but any kin present; (6) mother only, no kin present; and (7) other (e.g., lone father and other uncommon combinations). These categories were created based on the mother's identification number, which allowed us to describe the presence of matrilineal members in the household. Father linkages with kin are not as robust over time, given the focus of the DSSs on vital events such as fertility – our typology is therefore limited to matrilineal kin. For kinship co-residence, we created an indicator of grandmother, and counts of aunts, uncles, and older brothers and sisters (aged 5+). We also included an indicator of parental co-residence as both parents, one parent, or no parent resident in the household. All of the household structure and composition indicators were time-varying.

Statistical analysis

We organized the data as person-months for children under five years of age (0-59 months), where each child was at risk of dying for each month they were observed (up to and including death). We modelled mortality using discrete time event history analysis and used multi-level relative risk regression models to estimate a child's risk of dying.[26, 27]

To test for differences in household relationships between the two sites and over time, we included a binary site indicator (AHDSS or AHRI), and a time period indicator of 2000-2007 and 2008-2015. The split between 2007 and 2008 marks the time period when ART was available at both sites. At the household-level we included counts of the number of other household members ages 0-4, 5-19, and 20 years and older. We also included controls for child sex and age (<1 month, 1-6 months, 7-23 months, and 24-59 months); multiple birth (singleton or multiple); and mother's age at birth (15-19, 20-24, 25-29, 30-34, and 35+ years). We tested if the structural typologies and kinship and parental presence associations varied over time, between sites, and by child sex and age using nested likelihood ratio tests.

Finally, in a sub-model we included gender of household head and household socioeconomic status (SES) as important controls for our main finding. Household SES was based on a

For peer review only - http://bmjopen.bmj.com/site/about/guidelines.xhtml

common set of measures measured at each DSS since 2001, summarized using principal components analysis.[28] We used tertiles of the first principal component score from the most recent measurement. Given the truncated time span and higher levels of missing data on these two covariates, we used this sub-model as a sensitivity test to determine if these factors explained our main findings.

Patient and public involvement

Neither study participants nor public were involved in study design or conduct of the study. Both AHDSS and AHRI have ongoing liaison and open dialogue with the HDSS study communities and their leaders.

RESULTS

We first describe under-five mortality patterns by time and DSS. Out of a total of 101,105 children, 3,603 died between 2000-2015. Online supplemental figure 1 shows the mortality rate per 1,000 for children under 5 by year and DSS. Mortality began decreasing at AHRI in 2004 and AHDSS in 2009.

Household structure

Next, we examine differences between DSSs in the distribution of household structures over time (figure 1). For AHDSS, about 40% of households were nuclear only compared to a declining proportion of households at AHRI over time (33% in 2000 to 15% in 2015). Vertical and horizontal households represented about one-third of household structures at AHRI, while at AHDSS this household type has been increasing over time (15% in 2000 to 28% in 2015). Given the very low percentage of horizontal only households at AHDSS, in the subsequent mortality analysis we collapsed this group with vertical and horizontal households (initial tests indicated similar mortality patterns between these two groups). While rare at both DSSs, we preserved the no parents, kin present typology given its conceptual distinctiveness.

The results from the full household structure model are presented in online supplemental table 1. An interaction between DSS and time period ($p<0.001$), DSS and household structure ($p=0.001$), and time and household structure ($p=0.004$) significantly improved model fit. A

For peer review only

multi-level model including a mother random intercept improved model fit according to the Bayesian Information Criterion ($\Delta BIC = 156$) and resulted in the final model.

Figure 2 shows model-based predicted probabilities of a child dying by household structure, DSS, and time period. For both DSSs, the period of ART availability lowered the probability of dying across the different household types. For both DSSs, children in nuclear households had the lowest probability of dying compared to other household types. While most other household types had similar probabilities of dying, AHDSS children in mother only and other household types had elevated mortality risk, even in the ART period.

Kin presence

In developing a model for the presence of parents and kin, we tested several specifications including: counts vs. binary indicators of specific types of kin and testing for differences by child sex. Because we found that only related adults and older siblings had any association with child mortality, the results of the kin presence model in online supplemental table 2 present this more parsimonious categorization.

Figure 3 shows the proportion of children according to co-resident parents. Children living with no parents was 10% or lower at both DSSs, but has declined over time at AHDSS (11% in 2000 to 4% in 2015) while remaining relatively stable at AHRI (8% in 2000 and 2015). At both sites living with one parent was common and has steadily increased over time, but was increasingly more common at AHRI (58% in 2000 to 78% in 2015). Conversely, living with both parents remained relatively stable at AHDSS over time (44% in 2000 to 37% in 2015), but declined over time at AHRI (34% in 2000 to 15% in 2015). Comparing with household structure (Figure 3) also shows that the vast majority of children living in two parent households did not live with other related kin.

Figure 4 shows the proportion of children living with related adults and siblings (not mutually exclusive). The proportion of children living with related adults increased over time by about 20% at AHRI and 15% at AHDSS, and was more common for AHRI children. Living with siblings remained relatively stable over time at just under 50% at both DSSs.

The results from the full parent and kin presence model are presented in online supplemental table 2. An interaction between DSS and time period (p<0.001), parent co-residence and

For peer review only - http://bmjopen.bmj.com/site/about/guidelines.xhtml

related adults (p<0.001), parent co-residence and older siblings (p<0.001), and related adults and time period (p=0.003) significantly improved model fit. A multi-level model including a random intercept for the mother improved model fit according to the BIC ($\Delta BIC = 152$) and resulted in the final model.

Figure 5 shows the model-based predicted probabilities of a child dying by kin presence, parent co-residence, and time period. Having older siblings lowered the probability of dying only for children in a household with both parents (RRR=0.736 95% CI [0.633, 0.855]). While rare, having other adult kin present in two parent households resulted in a higher probability of dying for children. Only in the later ART period was there evidence that older adult kin lowered the probability of dying in single parent households (RRR=0.753 95% CI [0.664, 0.853]).

Sensitivity analysis

In the sub-models including household SES and household head gender, the main findings of household structure and composition remained (see online supplemental tables 3 and 4). Children in the highest (wealthiest) SES tertile had a lower risk of dying compared to children in the lowest (poorest) tertile.

DISCUSSION

Over a 16-year period, we examined associations between household structure and the role of kin in the household and differential mortality risk for children. Our main finding was that children in nuclear households with both parents had the lowest risk of dying. The kin presence model supported this finding, showing that having both parents as members of the household provided a protective effect for children.

The role of both parents is important to consider in light of the local context at both sites. Labour migration is common in both populations, and remittances play a beneficial role supporting children remaining in rural areas.[29] The protective role of the father has been shown in another comparative study from these sites,[2] highlighting their importance as long as they remain a breadwinner for the household.[30] However, our indicator of father presence does not capture the detail of their role and support in the household, particularly for those fathers non-coresident in the household.[31, 32] Female migration is also increasing over time,[33]

For peer review only - http://bmjopen.bmj.com/site/about/guidelines.xhtml

For peer review only

with resulting changes in the associations with child mortality.[30] Most children remain residents of their migrating parent's origin household.[34] Further, the availability of government pensions allows children of migrants to stay with grandmothers who have resources to support them.

We showed that associations with kin and child mortality were moderated by parental status. We found a protective effect of related adults in one parent households only in the period of widespread ART availability. The lack of a protective effect in the earlier HIV period likely reflects complex household dynamics given excess AIDS mortality in prime aged adults.[23, 25] For instance, one parent households during this time period were more likely to have experienced loss of a parent. Related adults may have also placed additional burden on the household in this configuration,[35] for instance if they were unwell themselves. With widespread ART availability, the likelihood of parental loss and unwell related adults would both be reduced, which may reflect these adults being able to now provide caretaking and other roles that support vulnerable children.[16, 36] Presence of related adults may also help by reducing resource strain when funds are directed to unwell household members and providing care for children when mothers become very ill.[2]

Our findings suggest heterogeneity in the configuration of households and their associations with child mortality between the two sites. Household diversity has changed over time, in part due to low marriage rates, high levels of labour migration and unemployment, and high HIV prevalence.[37, 38] Results from reviews on the role of kin on child mortality have shown between study heterogeneity in kin effects, due in part to different study designs and lack of consistent controls.[39, 40] A strength of our study is the use of harmonized data and a unified statistical framework to examine differences in kin associations between the two sites. Our results provide evidence for the supportive role of kin, dependent on different ecological and epidemiological conditions that vary over time and between local contexts.

We acknowledge study limitations. First, our household variables only include matrilineal kin given the greater consistency over time of maternal identifiers. To create a harmonized data structure across the two sites, we also only identified household structure and composition based on memberships information. We therefore lack detailed information on the roles of matrilineal kin on child caretaking and nutrition, and how this may vary by parental status. Formative research is needed to understand the role of other household kin, particularly in

For peer review only - http://bmjopen.bmj.com/site/about/guidelines.xhtml

one-parent households. Using household memberships also means that we cannot account for the role of kin who may provide child caretaking or support but are members of another household. Associations between household structure and composition may also be due to other unmeasured risk factors. We used multi-level modelling to account for shared mortality risk for children in the same household. Our results were also robust to controls for household SES and household head gender.

A key contribution of this study was to provide comparative, longitudinal evidence on associations between household structure and composition with child mortality from two rural South African populations both heavily burdened by HIV/AIDS. With the rollout of ART and rapid changes in SES,[23, 25, 28] these settings are continuing to undergo rapid demographic, epidemiological, and social transitions. Further longitudinal research is needed to understand continued changes in living arrangements and the role of parents and kin in protecting the well-being of children.

For peer review only

Ethics approval

Ethics approval for AHDSS was obtained from the Human Research Ethics Committee (Medical) of the University of the Witwatersrand, Johannesburg, South Africa (protocols M960720 and M110138). Ethics approval for AHRI was obtained from the Biomedical Research Ethics Committee (BREC) of the University of KwaZulu-Natal, Durban, South Africa (reference number BE169/15).

Data availability statement

The data underlying this article will be shared on reasonable request to the corresponding author. The data underlying the results presented in the study are available from the AHRI Data Repository (https://data.africacentre.ac.za) for researchers who meet the criteria for access to confidential data and sign on the agreement according to the AHRI's policy for data sharing. Detailed documentation of the AHDSS data and an anonymized database containing data from 10% of the surveillance households are available for public access on the AHDSS website (http://www.agincourt.za). The AHDSS core demographic data are also routinely deposited for public access in the INDEPTH Network Data Repository (http://www.indepthishare.org/) and the SAPRIN Data Repository (http://saprindata.samrc.ac.za/index.php/catalog). Customized data extraction can be requested from Dr. F. Xavier Gómez-Olivé (F.Gomez-OliveCasas@wits.ac.za).

Funding

The authors received no specific funding for this study. The MRC/Wits Rural Public Health and Health Transitions Research Unit and Agincourt Health and Socio-Demographic Surveillance System, a node of the South African Population Research Infrastructure Network (SAPRIN), is supported by the Department of Science and Innovation, the University of the Witwatersrand, and the Medical Research Council, South Africa, and previously the Wellcome Trust, UK (grants 058893/Z/99/A; 069683/Z/02/Z; 085477/Z/08/Z; 085477/B/08/Z). The Africa Health Research Institute is supported largely by Wellcome Trust (grant 201433/Z/16/Z) and Howard Hughes Medical Institute. The population surveillance, a node of the South African Population Research Infrastructure Network (SAPRIN), is funded by the Department of Science and Innovation and hosted by the South African Medical Research Council.

Patient consent for publication

For peer review only

Not required.

Competing interests

None declared.

Contributors

BH and CWK conceived the study. BH wrote the first draft and designed and completed the statistical analyses. CWK prepared the data with the support of DG. KH and SJC provided overall guidance to the conduct of the study. CWK, DG, KH, and SJC revised the manuscript for important intellectual content and contributed to interpretation of the data. All authors read and approved the final manuscript.

For peer review only

REFERENCES

1. UN Inter-Agency Group for Child Mortality Estimation. Levels and Trends in Child Mortality: Report 2019. 2019. Available from: https://www.unicef.org/sites/default/files/2019-10/UN-IGME-child-mortality-report-2019.pdf
2. Houle B, Kabudula C, Stein A, Gareta D, Herst K, Clark S. Linking the timing of a mother’s and child’s death: Comparative evidence from two rural South African population-based surveillance studies, 2000-2015. *PLoS One* 2021;16:e0246671.
3. Tlou B, Sartorius B, Tanser F. Investigating risk factors for under-five mortality in an HIV hyper-endemic area of rural South Africa, from 2000-2014. *PLOS ONE* 2018;13:e0207294.
4. Zaba B, Whitworth J, Marston M et al. HIV and Mortality of Mothers and Children. *Epidemiology* 2005;16:275-280.
5. Ndirangu J, Newell ML, Tanser F, Herbst AJ, Bland R. Decline in early life mortality in a high HIV prevalence rural area of South Africa: evidence of HIV prevention or treatment impact? *AIDS* 2010;24:593-602.
6. Ndirangu J, Newell ML, Thorne C, Bland R. Treating HIV-infected mothers reduces under 5 years of age mortality rates to levels seen in children of HIV-uninfected mothers in rural South Africa. *Antivir Ther* 2012;17:81-90.
7. Newell M-L, Brahmbhatt H, Ghys PD. Child mortality and HIV infection in Africa: a review. *AIDS* 2004;18:S27-S34.
8. Desmond C, Michael K, Gow J. The Hidden Battle : HIV / AIDS in the Family and Community. *South African Journal of International Affairs* 2000;7:39-58.
9. Madhavan S, Townsend N. The social context of children’s nutritional status in rural South Africa. *Scand J Public Health Suppl* 2007;69:107-117.
10. Madhavan S. Fosterage patterns in the age of AIDS: continuity and change. *Social science & medicine* 2004;58:1443-1454.
11. Schatz EJ. “Taking care of my own blood”: older women’s relationships to their households in rural South Africa. *Scand J Public Health Suppl* 2007;69:147-154.
12. Schatz E, Ogunmefun C. Caring and Contributing: The Role of Older Women in Rural South African Multi-generational Households in the HIV/AIDS Era. *World Development* 2007;35:1390-1403.
13. Gibson MA, Mace R. Helpful grandmothers in rural Ethiopia: A study of the effect of kin on child survival and growth. *Evolution and Human Behavior* 2005;26:469-482.
14. Omariba WR, Rajulton F, Beaujot R. Correlated mortality risks of siblings in Kenya. *Demographic Research* 2008;18:311-336.
15. Ronsmans C. Patterns of Clustering of Child Mortality in a Rural Area of Senegal. *Population Studies* 1995;49:443-461.
16. Sear R, Steele F, McGregor IA, Mace R. The effects of kin on child mortality in rural Gambia. *Demography* 2002;39:43-63.
17. van Dijk IK. Early-life mortality clustering in families: A literature review. *Popul Stud (Camb)* 20181-21.
18. Øyen N, Boyd HA, Poulsen G, Wohlfahrt J, Melbye M. The clustering of premature deaths in families. *Epidemiology* 2009;20:757-765.
19. Houle B, Stein A, Kahn K et al. Household context and child mortality in rural South Africa: the effects of birth spacing, shared mortality, household composition and socio-economic status. *Int J Epidemiol* 2013;42:1444-1454.

For peer review only

For peer review only - http://bmjopen.bmj.com/site/about/guidelines.xhtml

20. Horton R. The continuing invisibility of women and children. *The Lancet* 2010;375:1941-1943.
21. Madhavan S, Myroniuk TW, Kuhn R, Collinson MA. Household structure vs. composition: Understanding gendered effects on educational progress in rural South Africa. *Demogr Res* 2017;37:1891-1916.
22. Kahn K, Collinson MA, Gomez-Olive FX et al. Profile: Agincourt Health and Socio-demographic Surveillance System. *Int J Epidemiol* 2012;41:988-1001.
23. Kabudula C, Houle B, Ohene-Kwofie D et al. Mortality transition over a quarter century in rural South Africa: Finding from population surveillance in Agincourt 1993 — 2018. *Global Health Action* 2021;14:1990507.
24. Gareta D, Baisley K, M'ngomezulu T et al. Cohort profile update: Africa Centre Demographic Information System (ACDIS) and population-based HIV survey. *International Journal of Epidemiology* 202133-34f.
25. Reniers G, Blom S, Calvert C et al. Trends in the burden of HIV mortality after roll-out of antiretroviral therapy in KwaZulu-Natal, South Africa: an observational community cohort study. *The Lancet HIV* 2017;4:e113-e121.
26. Allison PD. Discrete-Time Methods for the Analysis of Event Histories. *Sociological Methodology* 1982;13:61-98.
27. Lumley T, Kronmal R, Ma S. Relative risk regression in medical research: Models, contrasts, estimators, and algorithms. *UW Biostatistics Working Paper Series* 2006;293
28. Kabudula C, Houle B, Collinson MA, Kahn K, Tollman S, Clark S. Assessing changes in household socioeconomic status in rural South Africa, 2001-2013: a distributional analysis using household asset indicators. *Social Indicators Research* 2017;133:1047-1073.
29. Collinson M. The Dynamics of Migration, Health and Livelihoods: INDEPTH Network Perspectives. 2009
30. Collinson MA. Striving against adversity: the dynamics of migration, health and poverty in rural South Africa. *Global Health Action* 2010;3:1-15.
31. Clark S, Cotton C, Marteleto LJ. Family Ties and Young Fathers' Engagement in Cape Town, South Africa. *Journal of Marriage and Family* 2015;77:575-589.
32. Madhavan S, Townsend NW, Garey AI. 'Absent Breadwinners': Father-Child Connections and Paternal Support in Rural South Africa. *J South Afr Stud* 2008;34:647-663.
33. Collinson MA, Tollman SM, Kahn K. Migration, settlement change and health in post-apartheid South Africa: Triangulating health and demographic surveillance with national census data1. *Scandinavian Journal of Public Health* 2007;35:77-84.
34. Bennett R, Hosegood V, Newell M, McGrath N. An approach to measuring dispersed families with a particular focus on children 'left behind'by migrant parents: findings from rural South Africa. *Population, space and place* 2015;21:322-334.
35. Basaza R, Kaija D, Ochola-Odonga D. The impact of HIV/AIDS on children: Lights and shadows in the "Successful Case" of Uganda. *AIDS, public policy and child well-being* 200731-66.
36. Cunningham SA, Elo IT, Herbst K, Hosegood V. Prenatal development in rural South Africa: relationship between birth weight and access to fathers and grandparents. *Popul Stud (Camb)* 2010;64:229-246.
37. Hosegood V. The demographic impact of HIV and AIDS across the family and household life-cycle: implications for efforts to strengthen families in sub-Saharan Africa. *AIDS Care* 2009;21 Suppl 1:13-21.
38. Madhavan S, Schatz EJ. Coping with change: household structure and composition in rural South Africa, 1992 - 2003. *Scand J Public Health Suppl* 2007;69:85-93.

For peer review only - http://bmjopen.bmj.com/site/about/guidelines.xhtml

39. Sear R, Mace R. Who keeps children alive? A review of the effects of kin on child survival. *Evolution and Human Behavior* 2008;29:1-18.
40. Strassmann BI, Garrard WM. Alternatives to the grandmother hypothesis: a meta-analysis of the association between grandparental and grandchild survival in patrilineal populations. *Hum Nat* 2011;22:201-222.

For peer review only

FIGURE CAPTIONS

Figure 1. Distribution of household structures over time and demographic surveillance site, Agincourt Health and Demographic Surveillance System (AHDSS) and Africa Health Research Institute (AHRI), South Africa 2000-2015.

Figure 2. Monthly probability of child death, by household structure and demographic surveillance site: Agincourt Health and Demographic Surveillance System (AHDSS) and Africa Health Research Institute (AHRI), South Africa 2000-2015. Jittered points to reduce over plotting.

Figure 3. Distribution of parent co-residence with children over time and demographic surveillance site, Agincourt Health and Demographic Surveillance System (AHDSS) and Africa Health Research Institute (AHRI), South Africa 2000-2015.

Figure 4. Proportion of children living with related adults and siblings (not mutually exclusive) over time and demographic surveillance site, Agincourt Health and Demographic Surveillance System (AHDSS) and Africa Health Research Institute (AHRI), South Africa 2000-2015.

Figure 5. Monthly probability of child death, by parent co-residence, kin presence, and time period: Agincourt Health and Demographic Surveillance System (AHDSS) and Africa Health Research Institute (AHRI), South Africa 2000-2015. Jittered points to reduce over plotting.

For peer review only

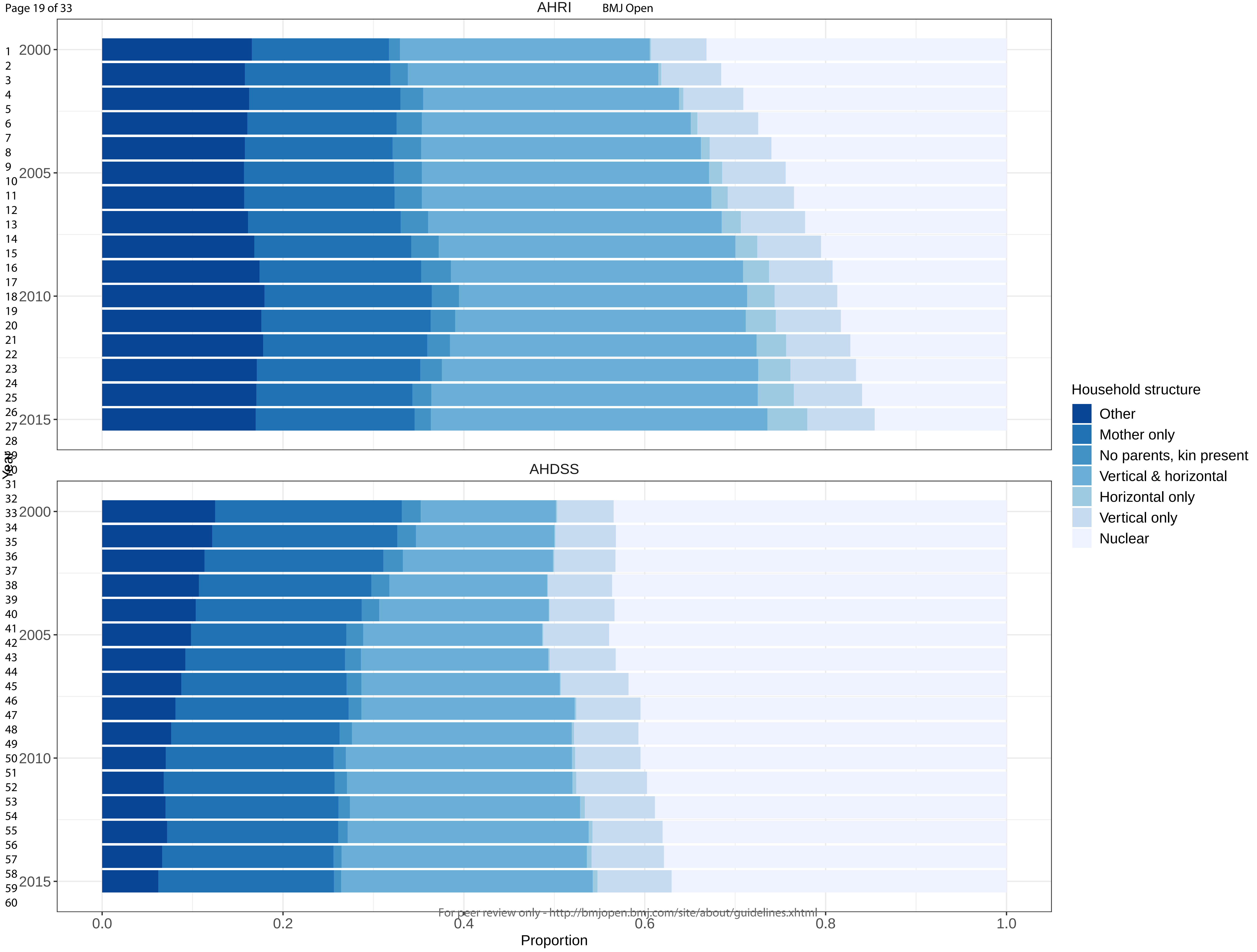

For peer review only - http://bmjopen.bmj.com/site/about/guidelines.xhtml

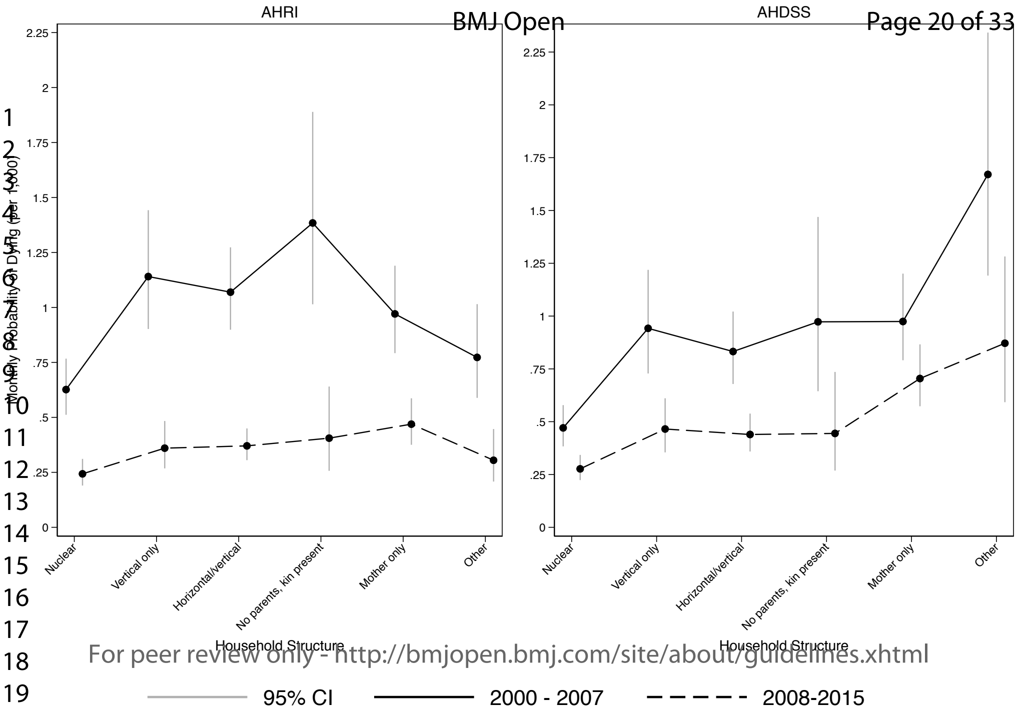

For peer review only - http://bmjopen.bmj.com/site/about/guidelines.xhtml

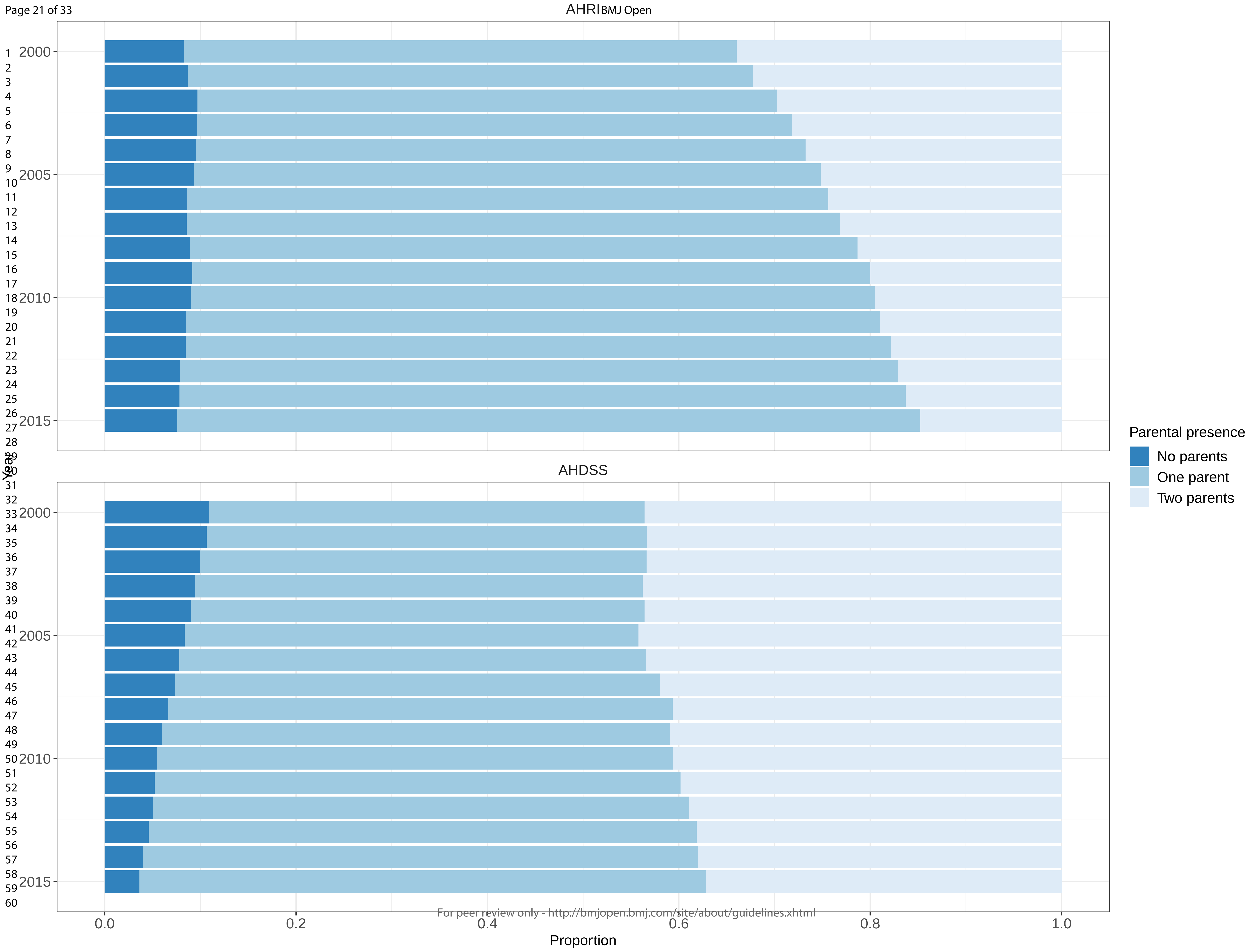

For peer review only - http://bmjopen.bmj.com/site/about/guidelines.xhtml

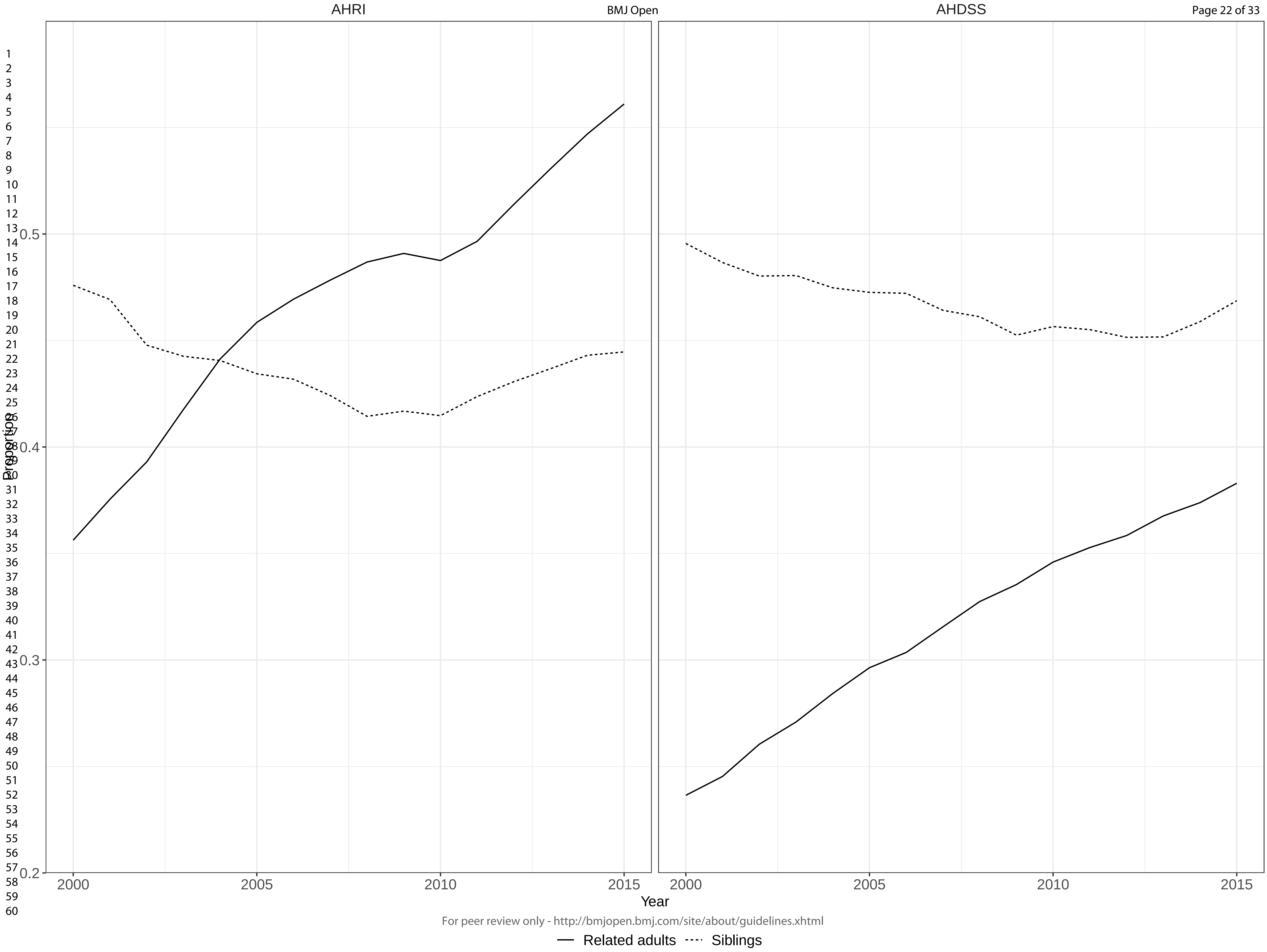
AHRI
AHDSS
Proportion
0.5
0.4
0.3
0.2
2000
2005
2010
2015
Year
Related adults
Siblings

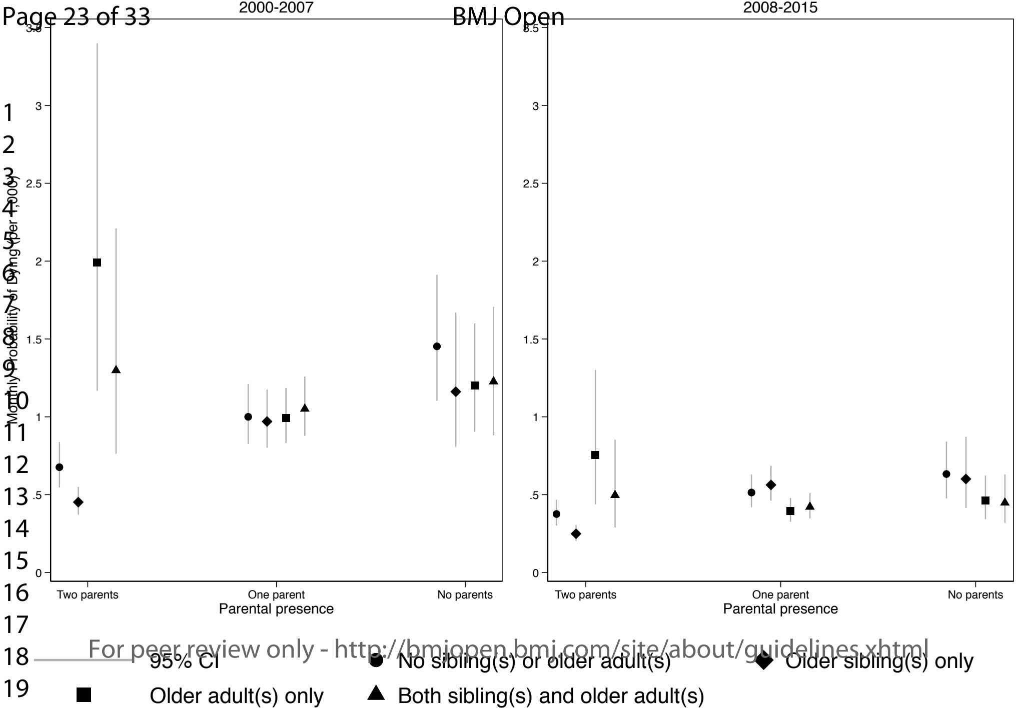
2000-2007
2008-2015
Monthly Probability of Dying (per 1,000)
0
.5
1
1.5
2
2.5
3
Two parents
One parent
No parents
Parental presence
95% CI
No sibling(s) or older adult(s)
Older sibling(s) only
Older adult(s) only
Both sibling(s) and older adult(s)

Supplemental Figure 1. Child mortality rates by year, Agincourt Health and Demographic Surveillance System (AHDSS) and Africa Health Research Institute (AHRI), South Africa 2000-2015.

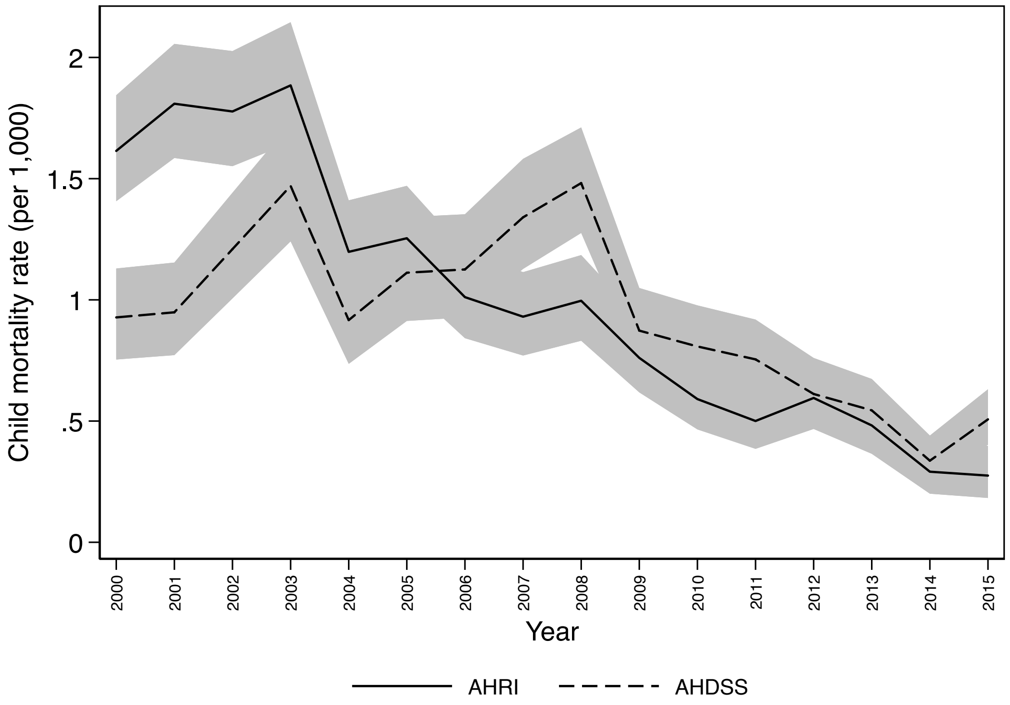

Supplemental Table 1. Multilevel relative risk regression of child death on household structure and controls, Agincourt Health and Demographic Surveillance System (AHDSS) and Africa Health Research Institute (AHRI), South Africa 2000-2015 (n=3,637,638 child months).

	RRR	95% CI	p-value
Site			
AHRI	1	-	
AHDSS	0.757	[0.647, 0.887]	0.001
Time period			
2000 to 2007	1	-	
2008 to 2015	0.415	[0.346, 0.498]	<0.001
Site X time period			
AHDSS X 2008 to 2015	1.454	[1.251, 1.689]	<0.001
Sex of child			
Female	1	-	
Male	1.124	[1.049, 1.204]	0.001
Child age (months)			
<1	1	-	
1 to 6	0.38	[0.342, 0.423]	<0.001
7 to 23	0.177	[0.159, 0.196]	<0.001
24 to 59	0.035	[0.031, 0.039]	<0.001
Multiple birth			
Singleton	1	-	
Multiple birth	1.818	[1.557, 2.124]	<0.001
Mother's age at birth (years			
15-19	0.864	[0.780, 0.956]	0.005
20-24	1	-	
25-29	1.264	[1.148, 1.392]	<0.001
30-34	1.089	[0.969, 1.225]	0.152
35+	1.237	[1.091, 1.403]	0.001
Number ages 0-5			
0	1	-	
1	1.098	[1.011, 1.192]	0.027
2+	1.251	[1.138, 1.376]	<0.001
Number ages 5-19			
0	1	-	
1 to 2	0.98	[0.849, 1.132]	0.787
3 to 4	0.928	[0.800, 1.076]	0.321
5+	1.044	[0.894, 1.219]	0.588
Number ages 20+			
0 to 2	1	-	
3 to 4	0.975	[0.879, 1.081]	0.631
5+	0.938	[0.841, 1.046]	0.249

For peer review only

Household structure			
Nuclear	1	-	
Vertical only	1.644	[1.325, 2.041]	<0.001
Horizontal/vertical	1.635	[1.408, 1.898]	<0.001
No parents, kin present	2.906	[2.169, 3.895]	<0.001
Mother only	1.554	[1.313, 1.839]	<0.001
Other	1.809	[1.411, 2.319]	<0.001
Site X household structure			
AHDSS X Vertical only	1.181	[0.892, 1.563]	0.245
AHDSS X Horizontal/vertical	1.07	[0.877, 1.305]	0.506
AHDSS X No parents, kin present	0.938	[0.595, 1.479]	0.784
AHDSS X Mother only	1.373	[1.112, 1.696]	0.003
AHDSS X Other	1.932	[1.339, 2.787]	<0.001
Time period X household structure			
2008 to 2015 X Vertical only	0.842	[0.632, 1.122]	0.241
2008 to 2015 X Horizontal/vertical	0.905	[0.742, 1.104]	0.324
2008 to 2015 X No parents, kin present	0.818	[0.506, 1.323]	0.413
2008 to 2015 X Mother only	1.2	[0.973, 1.479]	0.089
2008 to 2015 X Other	1.07	[0.725, 1.578]	0.734
	Parameter		
σ^2_{mother}	3.152	[2.574, 3.859]	

For peer review only

Supplemental Table 2. Multilevel relative risk regression of child death on kin presence and controls, Agincourt Health and Demographic Surveillance System (AHDSS) and Africa Health Research Institute (AHRI), South Africa 2000-2015 (n=3,637,638 child months).

	RRR	95% CI	p-value
Site			
AHRI	1	-	
AHDSS	0.874	[0.794, 0.961]	0.006
Time period			
2000 to 2007	1	-	
2008 to 2015	0.456	[0.399, 0.520]	<0.001
Site X time period			
AHDSS X 2008 to 2015	1.437	[1.241, 1.664]	<0.001
Sex of child			
Female	1	-	
Male	1.125	[1.050, 1.205]	0.001
Child age (months)			
<1	1	-	
1 to 6	0.379	[0.341, 0.422]	<0.001
7 to 23	0.176	[0.159, 0.195]	<0.001
24 to 59	0.035	[0.031, 0.039]	<0.001
Multiple birth			
Singleton	1	-	
Multiple birth	1.804	[1.545, 2.106]	<0.001
Mother's age at birth (years			
15-19	0.866	[0.780, 0.961]	0.007
20-24	1	-	
25-29	1.277	[1.155, 1.413]	<0.001
30-34	1.127	[0.995, 1.278]	0.06
35+	1.309	[1.143, 1.499]	<0.001
Number ages 0-5			
0	1	-	
1	1.096	[1.009, 1.190]	0.029
2+	1.249	[1.136, 1.373]	<0.001
Number ages 5-19			
0	1	-	
1 to 2	1.069	[0.917, 1.246]	0.395
3 to 4	1.014	[0.864, 1.189]	0.865
5+	1.147	[0.969, 1.357]	0.11
Number ages 20+			
0 to 2	1	-	
3 to 4	0.953	[0.861, 1.056]	0.359
5+	0.903	[0.811, 1.004]	0.06
Parental presence			

For peer review only

Both parents	1	-	
One parent	1.569	[1.363, 1.807]	<0.001
Neither parent	2.623	[2.058, 3.343]	<0.001
Related adult presence			
No	1	-	
Yes	2.954	[1.769, 4.931]	<0.001
Parental presence X related adult presence			
One parent X related adult present	0.316	[0.188, 0.530]	<0.001
Neither parent X related adult present	0.307	[0.171, 0.551]	<0.001
Related siblings			
No	1	-	
Yes	0.712	[0.606, 0.837]	<0.001
Parental presence X related siblings			
One parent X related siblings present	1.464	[1.233, 1.737]	<0.001
Neither parent X related siblings present	1.518	[1.070, 2.153]	0.019
Time period X related adult presence			
2008 to 2015 X related adult presence	0.806	[0.698, 0.932]	0.004
	Parameter		
σ^2_{mother}	1.128	[0.944, 1.349]	

For peer review only

Supplemental Table 3. Multilevel relative risk regression of child death on household structure and controls, Agincourt Health and Demographic Surveillance System (AHDSS) and Africa Health Research Institute (AHRI), South Africa 2000-2015 (n=3,637,638 child months). Estimation sample restricted to those with household SES information.

	RRR	95% CI	p-value
Site			
AHRI	1	-	
AHDSS	0.772	[0.625, 0.954]	0.017
Time period			
2000 to 2007	1	-	
2008 to 2015	0.391	[0.305, 0.501]	<0.001
Site X time period			
AHDSS X 2008 to 2015	1.395	[1.141, 1.705]	0.001
Sex of child			
Female	1	-	
Male	1.134	[1.037, 1.240]	0.006
Child age (months)			
<1	1	-	
1 to 6	0.355	[0.304, 0.415]	<0.001
7 to 23	0.176	[0.152, 0.205]	<0.001
24 to 59	0.036	[0.030, 0.042]	<0.001
Multiple birth			
Singleton	1	-	
Multiple birth	1.58	[1.281, 1.948]	<0.001
Mother's age at birth (years			
15-19	0.897	[0.785, 1.024]	0.107
20-24	1	-	
25-29	1.364	[1.202, 1.548]	<0.001
30-34	1.175	[1.008, 1.368]	0.039
35+	1.391	[1.183, 1.634]	<0.001
Number ages 0-5			
0	1	-	
1	1.049	[0.943, 1.167]	0.375
2+	1.225	[1.082, 1.387]	0.001
Number ages 5-19			
0	1	-	
1 to 2	1.031	[0.855, 1.243]	0.746
3 to 4	0.949	[0.782, 1.152]	0.594
5+	1.034	[0.843, 1.268]	0.748
Number ages 20+			
0 to 2	1	-	
3 to 4	0.912	[0.798, 1.041]	0.174
5+	0.868	[0.753, 1.002]	0.053

For peer review only

Household structure			
Nuclear	1	-	
Vertical only	1.851	[1.374, 2.495]	<0.001
Horizontal/vertical	1.733	[1.408, 2.131]	<0.001
No parents, kin present	3.356	[2.292, 4.914]	<0.001
Mother only	1.716	[1.357, 2.170]	<0.001
Other	1.892	[1.335, 2.680]	<0.001
Site X household structure			
AHDSS X Vertical only	1.099	[0.752, 1.604]	0.627
AHDSS X Horizontal/vertical	1.127	[0.863, 1.470]	0.38
AHDSS X No parents, kin present	1.072	[0.619, 1.857]	0.804
AHDSS X Mother only	1.309	[0.981, 1.746]	0.067
AHDSS X Other	2.069	[1.274, 3.360]	0.003
Time period X household structure			
2008 to 2015 X Vertical only	0.734	[0.502, 1.075]	0.112
2008 to 2015 X Horizontal/vertical	0.838	[0.648, 1.085]	0.181
2008 to 2015 X No parents, kin present	0.763	[0.416, 1.399]	0.382
2008 to 2015 X Mother only	1.278	[0.978, 1.669]	0.073
2008 to 2015 X Other	1.044	[0.624, 1.745]	0.87
Household SES			
Low	1	-	
Middle	0.949	[0.852, 1.058]	0.346
High	0.788	[0.702, 0.883]	<0.001
Household head gender			
Female	1	-	
Male	1.104	[0.999, 1.220]	0.052
	Parameter		
σ^2_{mother}	3.152	[2.574, 3.859]	

For peer review only

Supplemental Table 4. Multilevel relative risk regression of child death on kin presence and controls, Agincourt Health and Demographic Surveillance System (AHDSS) and Africa Health Research Institute (AHRI), South Africa 2000-2015 (n=2,468,466 child months). Estimation sample restricted to those with household SES information.

	RRR	95% CI	p-value
Site			
AHRI	1	-	
AHDSS	0.903	[0.797, 1.022]	0.106
Time period			
2000 to 2007	1	-	
2008 to 2015	0.442	[0.367, 0.532]	<0.001
Site X time period			
AHDSS X 2008 to 2015	1.374	[1.129, 1.672]	0.002
Sex of child			
Female	1	-	
Male	1.136	[1.039, 1.242]	0.005
Child age (months)			
<1	1	-	
1 to 6	0.354	[0.303, 0.414]	<0.001
7 to 23	0.176	[0.152, 0.204]	<0.001
24 to 59	0.036	[0.030, 0.042]	<0.001
Multiple birth			
Singleton	1	-	
Multiple birth	1.571	[1.275, 1.936]	<0.001
Mother's age at birth (years			
15-19	0.889	[0.775, 1.019]	0.091
20-24	1	-	
25-29	1.4	[1.227, 1.597]	<0.001
30-34	1.246	[1.059, 1.466]	0.008
35+	1.523	[1.280, 1.812]	<0.001
Number ages 0-5			
0	1	-	
1	1.046	[0.941, 1.163]	0.406
2+	1.219	[1.077, 1.380]	0.002
Number ages 5-19			
0	1	-	
1 to 2	1.19	[0.974, 1.454]	0.089
3 to 4	1.103	[0.894, 1.360]	0.36
5+	1.212	[0.971, 1.513]	0.089
Number ages 20+			
0 to 2	1	-	
3 to 4	0.89	[0.780, 1.015]	0.081
5+	0.827	[0.719, 0.951]	0.008

For peer review only

Parental presence			
Both parents	1	-	
One parent	1.609	[1.341, 1.930]	<0.001
Neither parent	2.865	[2.086, 3.933]	<0.001
Related adult presence			
No	1	-	
Yes	4.031	[2.137, 7.604]	<0.001
Parental presence X related adult presence			
One parent X related adult present	0.228	[0.120, 0.432]	<0.001
Neither parent X related adult present	0.229	[0.110, 0.474]	<0.001
Related siblings			
No	1	-	
Yes	0.6	[0.487, 0.739]	<0.001
Parental presence X related siblings			
One parent X related siblings present	1.695	[1.360, 2.113]	<0.001
Neither parent X related siblings present	1.84	[1.185, 2.856]	0.007
Time period X related adult presence			
2008 to 2015 X related adult presence	0.722	[0.599, 0.871]	0.001
Household SES			
Low	1	-	
Middle	0.946	[0.849, 1.054]	0.314
High	0.784	[0.699, 0.879]	<0.001
Household head gender			
Female	1	-	
Male	1.107	[1.004, 1.221]	0.042
	Parameter		
σ^2_{mother}	3.375	[2.494, 4.568]	

For peer review only

STROBE Statement—checklist of items that should be included in reports of observational studies

	Item No	Recommendation	Page No
**Title and abstract**	1	(*a*) Indicate the study's design with a commonly used term in the title or the abstract	1
		(*b*) Provide in the abstract an informative and balanced summary of what was done and what was found	2
**Introduction**			
Background/rationale	2	Explain the scientific background and rationale for the investigation being reported	4
Objectives	3	State specific objectives, including any prespecified hypotheses	4-5
**Methods**			
Study design	4	Present key elements of study design early in the paper	5
Setting	5	Describe the setting, locations, and relevant dates, including periods of recruitment, exposure, follow-up, and data collection	5
Participants	6	(*a*) *Cohort study*—Give the eligibility criteria, and the sources and methods of selection of participants. Describe methods of follow-up<br>*Case-control study*—Give the eligibility criteria, and the sources and methods of case ascertainment and control selection. Give the rationale for the choice of cases and controls<br>*Cross-sectional study*—Give the eligibility criteria, and the sources and methods of selection of participants	6
		(*b*) *Cohort study*—For matched studies, give matching criteria and number of exposed and unexposed<br>*Case-control study*—For matched studies, give matching criteria and the number of controls per case	
Variables	7	Clearly define all outcomes, exposures, predictors, potential confounders, and effect modifiers. Give diagnostic criteria, if applicable	6
Data sources/ measurement	8*	For each variable of interest, give sources of data and details of methods of assessment (measurement). Describe comparability of assessment methods if there is more than one group	5-7
Bias	9	Describe any efforts to address potential sources of bias	7
Study size	10	Explain how the study size was arrived at	6-7
Quantitative variables	11	Explain how quantitative variables were handled in the analyses. If applicable, describe which groupings were chosen and why	6-7
Statistical methods	12	(*a*) Describe all statistical methods, including those used to control for confounding	6-7
		(*b*) Describe any methods used to examine subgroups and interactions	6-7
		(*c*) Explain how missing data were addressed	6
		(*d*) *Cohort study*—If applicable, explain how loss to follow-up was addressed<br>*Case-control study*—If applicable, explain how matching of cases and controls was addressed<br>*Cross-sectional study*—If applicable, describe analytical methods taking account of sampling strategy	
		(*e*) Describe any sensitivity analyses	7

Continued on next page

For peer review only - http://bmjopen.bmj.com/site/about/guidelines.xhtml

**Results**			
Participants	13*	(a) Report numbers of individuals at each stage of study—eg numbers potentially eligible, examined for eligibility, confirmed eligible, included in the study, completing follow-up, and analysed	
		(b) Give reasons for non-participation at each stage	
		(c) Consider use of a flow diagram	
Descriptive data	14*	(a) Give characteristics of study participants (eg demographic, clinical, social) and information on exposures and potential confounders	Fig1,3,4
		(b) Indicate number of participants with missing data for each variable of interest	
		(c) *Cohort study*—Summarise follow-up time (eg, average and total amount)	
Outcome data	15*	*Cohort study*—Report numbers of outcome events or summary measures over time	
		Case-control study—Report numbers in each exposure category, or summary measures of exposure	
		Cross-sectional study—Report numbers of outcome events or summary measures	
Main results	16	(*a*) Give unadjusted estimates and, if applicable, confounder-adjusted estimates and their precision (eg, 95% confidence interval). Make clear which confounders were adjusted for and why they were included	
		(*b*) Report category boundaries when continuous variables were categorized	
		(*c*) If relevant, consider translating estimates of relative risk into absolute risk for a meaningful time period	
Other analyses	17	Report other analyses done—eg analyses of subgroups and interactions, and sensitivity analyses	9
**Discussion**			
Key results	18	Summarise key results with reference to study objectives	9
Limitations	19	Discuss limitations of the study, taking into account sources of potential bias or imprecision. Discuss both direction and magnitude of any potential bias	10-11
Interpretation	20	Give a cautious overall interpretation of results considering objectives, limitations, multiplicity of analyses, results from similar studies, and other relevant evidence	11
Generalisability	21	Discuss the generalisability (external validity) of the study results	10
**Other information**			
Funding	22	Give the source of funding and the role of the funders for the present study and, if applicable, for the original study on which the present article is based	12

*Give information separately for cases and controls in case-control studies and, if applicable, for exposed and unexposed groups in cohort and cross-sectional studies.

Note: An Explanation and Elaboration article discusses each checklist item and gives methodological background and published examples of transparent reporting. The STROBE checklist is best used in conjunction with this article (freely available on the Web sites of PLoS Medicine at http://www.plosmedicine.org/, Annals of Internal Medicine at http://www.annals.org/, and Epidemiology at http://www.epidem.com/). Information on the STROBE Initiative is available at www.strobe-statement.org.

For peer review only

BMJ Open

Household structure, composition, and child mortality in the unfolding antiretroviral therapy era in rural South Africa: Comparative evidence from population surveillance, 2000-2015

Journal:	*BMJ Open*
Manuscript ID	bmjopen-2022-070388.R1
Article Type:	Original research
Date Submitted by the Author:	09-Feb-2023
Complete List of Authors:	Houle , Brian ; Australian National University, School of Demography; University of the Witwatersrand School of Public Health, MRC/Wits Rural Public Health and Health Transitions Research Unit (Agincourt)<br>Kabudula, Chodziwadziwa; University of the Witwatersrand School of Public Health, MRC/Wits Rural Public Health and Health Transitions Research Unit (Agincourt),<br>Gareta, Dickman; Africa Health Research Institute, Statistics and Data<br>Herbst, Kobus; Africa Health Research Institute; DSI-MRC South African Population Research Infrastructure Network (SAPRIN)<br>Clark, Samuel J; The Ohio State University, Department of Sociology; University of the Witwatersrand School of Public Health, MRC/Wits Rural Public Health and Health Transitions Research Unit (Agincourt)
**Primary Subject Heading**:	Epidemiology
Secondary Subject Heading:	Global health, HIV/AIDS, Public health
Keywords:	Public health < INFECTIOUS DISEASES, Epidemiology < TROPICAL MEDICINE, HIV & AIDS < INFECTIOUS DISEASES

SCHOLARONE™
Manuscripts

I, the Submitting Author has the right to grant and does grant on behalf of all authors of the Work (as defined in the below author licence), an exclusive licence and/or a non-exclusive licence for contributions from authors who are: i) UK Crown employees; ii) where BMJ has agreed a CC-BY licence shall apply, and/or iii) in accordance with the terms applicable for US Federal Government officers or employees acting as part of their official duties; on a worldwide, perpetual, irrevocable, royalty-free basis to BMJ Publishing Group Ltd ("BMJ") its licensees and where the relevant Journal is co-owned by BMJ to the co-owners of the Journal, to publish the Work in this journal and any other BMJ products and to exploit all rights, as set out in our licence.

The Submitting Author accepts and understands that any supply made under these terms is made by BMJ to the Submitting Author unless you are acting as an employee on behalf of your employer or a postgraduate student of an affiliated institution which is paying any applicable article publishing charge ("APC") for Open Access articles. Where the Submitting Author wishes to make the Work available on an Open Access basis (and intends to pay the relevant APC), the terms of reuse of such Open Access shall be governed by a Creative Commons licence – details of these licences and which Creative Commons licence will apply to this Work are set out in our licence referred to above.

Other than as permitted in any relevant BMJ Author's Self Archiving Policies, I confirm this Work has not been accepted for publication elsewhere, is not being considered for publication elsewhere and does not duplicate material already published. I confirm all authors consent to publication of this Work and authorise the granting of this licence.

For peer review only

Household structure, composition, and child mortality in the unfolding antiretroviral therapy era in rural South Africa: Comparative evidence from population surveillance, 2000-2015

Brian Houle[1,2,3*]
Chodziwadziwa W Kabudula[2]
Dickman Gareta[4]
Kobus Herbst[4,5]
Samuel J Clark[2,3,6]

[*] Corresponding author: 146 Ellery Crescent, Acton, ACT 0200. brian.houle@anu.edu.au. +61 6125 7355.

1 School of Demography, The Australian National University, Canberra, Australia
2 MRC/Wits Rural Public Health and Health Transitions Research Unit (Agincourt), School of Public Health, Faculty of Health Sciences, University of the Witwatersrand, Johannesburg, South Africa
3 CU Population Center, Institute of Behavioral Science, University of Colorado at Boulder, Boulder, Colorado, USA
4 Africa Health Research Institute, KwaZulu-Natal, South Africa
5 DSI-MRC South African Population Research Infrastructure Network (SAPRIN), Durban, South Africa
6 Department of Sociology, The Ohio State University, Columbus, Ohio, USA

Word count: 2,874

For peer review only

ABSTRACT

Objectives: The structure and composition of the household has important influences on child mortality. However, little is known about these factors in HIV-endemic areas and how associations may change with the introduction and widespread availability of antiretroviral treatment (ART). We use comparative, longitudinal data from two demographic surveillance sites in rural South Africa (2000-2015) on mortality of children younger than five years (n=101,105).

Design: We use multilevel discrete time event history analysis to estimate children's probability of dying by their matrilineal residential arrangements. We also test if associations have changed over time with ART availability.

Setting: Rural South Africa.

Participants: Children younger than five years (n=101,105).

Results: 3,603 children died between 2000-2015. Mortality risks differed by co-residence patterns along with different types of kin present in the household. Children in nuclear households with both parents had the lowest risk of dying compared to all other household types. Associations with kin and child mortality were moderated by parental status. Having older siblings lowered the probability of dying only for children in a household with both parents (relative risk ratio (RRR)=0.736 95% CI [0.633, 0.855]). Only in the later ART period was there evidence that older adult kin lowered the probability of dying for children in single parent households (RRR=0.753 95% CI [0.664, 0.853]).

Conclusions: Our findings provide comparative evidence of how differential household profiles may place children at higher mortality risk. Formative research is needed to understand the role of other household kin in promoting child well-being, particularly in one-parent households that are increasingly prevalent.

Keywords: child mortality; household; HIV/AIDS; South Africa; longitudinal

For peer review only

Strengths and limitations of this study

- We provide 16 years of prospective, harmonized population data from two rural areas of South Africa heavily impacted by HIV/AIDS to analyse associations between household structure and composition with child mortality in a unified statistical framework.
- We used multi-level modelling to account for shared mortality risk for children in the same household.
- We were also able to adjust for potential confounding with socioeconomic status and household head gender.
- Our measure of household kin is based on household memberships, meaning we cannot account for the role of kin who are members of another household.
- Our study was only able to identify the presence of matrilineal members in the household given limitations with father linkages over time.

For peer review only

INTRODUCTION

Reducing preventable child mortality remains an urgent global priority and central Sustainable Development Goal. Goal 3 targets reducing under-five mortality to 25 per 1,000 live births by 2030. While many countries are on track or have already reached this goal, the remaining global mortality burden is uneven. The majority of global under-five deaths occur in sub-Saharan Africa.[1] A greater understanding of the social, cultural, and contextual determinants of child mortality is critical to reduce child mortality in the region.

In the context of the HIV epidemic, a number of studies in sub-Saharan Africa have examined factors that impact child mortality, such as parental survival, [2–4] and HIV infection and antiretroviral therapy.[5–7] Household studies have focused on the role of older household members, fostering, and orphanhood.[8–12] Less attention has been paid to other contextual risk factors, especially with mortality changes associated with the HIV epidemic and more recently with the availability of antiretroviral therapy (ART). While there is a substantial literature from low and middle-income countries on the importance of factors such as household composition and child mortality,[13–18] there remains limited longitudinal evidence from HIV-endemic areas.[3, 19] The availability of antiretroviral therapy (ART) may also have changed the relative importance of different risk factors and how they influence child mortality[6] – highlighting the need for longitudinal data examining risk factors over time.

The extent to which studies are generalisable across settings is also unclear, particularly given differences in factors such as household organization and available resources. There is a lack of comparative research within countries, particularly amongst disadvantaged populations.[20] Understanding if and how household risk factors are associated with child mortality between different settings has important implications for policies and interventions to reduce preventable child mortality. Comparative evidence is needed to inform these efforts.

Our primary aim is to investigate the relationship between a child's risk of dying and their household's structure and composition. We follow the conceptual approach proposed by Madhavan et al.[21] by examining two household-level dimensions: (1) structure, referring to the configuration of generations in the household and level of nucleation; and (2) composition, referring to the availability of specific kin that may be moderated by parental

For peer review only - http://bmjopen.bmj.com/site/about/guidelines.xhtml

presence. We use comparative, longitudinal data from 2000-2015 from two demographic surveillance sites (DSS) in rural South Africa to examine these factors. The time period covers when ART was unavailable and after widespread availability, allowing for an assessment of if and how associations between different risk factors and child mortality have changed over time with ART availability.

METHOD

Setting

We used household census data from 2000-2015 for two populations living in rural South Africa. Both DSSs monitor a geographically defined population over time and collect prospective information, including demographic indicators (e.g., mortality, fertility, migration) as well as household-level information and social indicators. The first, the Agincourt Health and socio-Demographic Surveillance System (AHDSS), has been conducting an annual census update of the population since 1992, updating vital events (births, deaths, migration) along with socio-demographic information.[22] AHDSS is located in the Ehlanseni district of Mpumalanga. The primary ethnic group is amaShangaan. The population under surveillance was approximately 90,000 people in 2011. Until recently child and adult mortality were increasing.[23] ART became available in 2008, with resulting substantial reductions in mortality.[23]

The second site, the Africa Health Research Institute (AHRI), started bi-annual census updates in 2000.[24] AHRI is located in the Umkanyakude district of KwaZulu-Natal. The population is almost entirely Zulu-speaking and comprised approximately 90,000 people in 2011. HIV/AIDS has had a significant impact on the population, but the availability of ART in 2004 significantly increased life expectancy in the population.[25]

Each site conducts a verbal autopsy (VA) for individuals who died during surveillance rounds. A trained fieldworker or nurse uses a standardized VA instrument to interview the closest living relative of the decedent to record signs and symptoms experienced before their death.

Household measures

For peer review only - http://bmjopen.bmj.com/site/about/guidelines.xhtml

Both DSSs update a roster of household members at each census update. To create a harmonized data structure across the sites, we used the household membership data (which included membership start and end dates) to identify members of the same household as the index child.

Our key measures were household structure and composition. We followed the categorisations of Madhavan et al.[21] as their analysis was based at AHDSS and included a complementary, cross-sectional data set with detailed kinship data. The structural typology included only those kin co-resident in the household: (1) both parents and no other kin (nuclear); (2) one or both parents and grandmother (vertical); (3) one or both parents, aunts/uncles (horizontal); (4) one or both parents, grandmother and aunts/uncles (vertical and horizontal); (5) no parents but any kin present; (6) mother only, no kin present; and (7) other (e.g., lone father and other uncommon combinations). These categories were created based on the mother's identification number, which allowed us to describe the presence of matrilineal members in the household. Father linkages with kin are not as robust over time, given the focus of the DSSs on vital events such as fertility – our typology is therefore limited to matrilineal kin. For kinship co-residence, we created an indicator of grandmother, and counts of aunts, uncles, and older brothers and sisters (aged 5+). We also included an indicator of parental co-residence as both parents, one parent, or no parent resident in the household. All of the household structure and composition indicators were time-varying.

Statistical analysis

We organized the data as person-months for children under five years of age (0-59 months), where each child was at risk of dying for each month they were observed (up to and including death). We modelled mortality using discrete time event history analysis and used multi-level relative risk regression models to estimate a child's risk of dying.[26, 27]

To test for differences in household relationships between the two sites and over time, we included a binary site indicator (AHDSS or AHRI), and a time period indicator of 2000-2007 and 2008-2015. The split between 2007 and 2008 marks the time period when ART was available at both sites. At the household-level we included counts of the number of other household members ages 0-4, 5-19, and 20 years and older. We also included controls for child sex and age (<1 month, 1-6 months, 7-23 months, and 24-59 months); multiple birth (singleton or multiple); and mother's age at birth (15-19, 20-24, 25-29, 30-34, and 35+

For peer review only - http://bmjopen.bmj.com/site/about/guidelines.xhtml

years). We tested if the structural typologies and kinship and parental presence associations varied over time, between sites, and by child sex and age using nested likelihood ratio tests.

We used InterVA-5 to assign causes of death.[28] InterVA is a model that assigns up to three causes of death based on the VA interview data – we used the cause of death with the largest likelihood for each complete VA interview. This allowed us to compare how the distribution of causes of death changed over time within each DSS.

Finally, in a sub-model we included gender of household head and household socioeconomic status (SES) as important controls for our main finding. Household SES was based on a common set of measures measured at each DSS since 2001, summarized using principal components analysis.[29] We used tertiles of the first principal component score from the most recent measurement. Given the truncated time span and higher levels of missing data on these two covariates, we used this sub-model as a sensitivity test to determine if these factors explained our main findings.

Patient and public involvement

Neither study participants nor public were involved in study design or conduct of the study. Both AHDSS and AHRI have ongoing liaison and open dialogue with the HDSS study communities and their leaders.

RESULTS

We first describe under-five mortality patterns by time and DSS. Out of a total of 101,105 children, 3,603 died between 2000-2015. Online supplemental figure 1 shows the mortality rate per 1,000 for children under 5 by year and DSS. Mortality began decreasing at AHRI in 2004 and AHDSS in 2009. Online supplemental figure 2 shows the distribution of child cause of death over the two time periods by DSS. For both AHRI and AHDSS, the share of deaths due to HIV/AIDS and TB declined in 2008-2015 compared to 2000-2007, while the share of deaths due to respiratory infections has increased over time.

Household structure

Next, we examine differences between DSSs in the distribution of household structures over time (figure 1). For AHDSS, about 40% of households were nuclear only compared to a

For peer review only - http://bmjopen.bmj.com/site/about/guidelines.xhtml

declining proportion of households at AHRI over time (33% in 2000 to 15% in 2015). Vertical and horizontal households represented about one-third of household structures at AHRI, while at AHDSS this household type has been increasing over time (15% in 2000 to 28% in 2015). Given the very low percentage of horizontal only households at AHDSS, in the subsequent mortality analysis we collapsed this group with vertical and horizontal households (initial tests indicated similar mortality patterns between these two groups). While rare at both DSSs, we preserved the no parents, kin present typology given its conceptual distinctiveness.

The results from the full household structure model are presented in online supplemental table 1. An interaction between DSS and time period ($p<0.001$), DSS and household structure ($p=0.001$), and time and household structure ($p=0.004$) significantly improved model fit. A multi-level model including a mother random intercept improved model fit according to the Bayesian Information Criterion ($\Delta BIC = 156$) and resulted in the final model. Twins, boys, younger children, and children in households with two or more other children have higher odds of mortality.

Figure 2 shows model-based predicted probabilities of a child dying by household structure, DSS, and time period. For both DSSs, the period of ART availability lowered the probability of dying across the different household types. For both DSSs, children in nuclear households had the lowest probability of dying compared to other household types. While most other household types had similar probabilities of dying, AHDSS children in mother only and other household types had elevated mortality risk, even in the ART period.

Kin presence

In developing a model for the presence of parents and kin, we tested several specifications including: counts vs. binary indicators of specific types of kin and testing for differences by child sex. Because we found that only related adults and older siblings had any association with child mortality, the results of the kin presence model in online supplemental table 2 present this more parsimonious categorization.

Figure 3 shows the proportion of children according to co-resident parents. Children living with no parents was 10% or lower at both DSSs, but has declined over time at AHDSS (11% in 2000 to 4% in 2015) while remaining relatively stable at AHRI (8% in 2000 and 2015). At

For peer review only - http://bmjopen.bmj.com/site/about/guidelines.xhtml

both sites living with one parent was common and has steadily increased over time, but was increasingly more common at AHRI (58% in 2000 to 78% in 2015). Conversely, living with both parents remained relatively stable at AHDSS over time (44% in 2000 to 37% in 2015), but declined over time at AHRI (34% in 2000 to 15% in 2015). Comparing with household structure (Figure 3) also shows that the vast majority of children living in two parent households did not live with other related kin.

Figure 4 shows the proportion of children living with related adults and siblings (not mutually exclusive). The proportion of children living with related adults increased over time by about 20% at AHRI and 15% at AHDSS, and was more common for AHRI children. Living with siblings remained relatively stable over time at just under 50% at both DSSs.

The results from the full parent and kin presence model are presented in online supplemental table 2. An interaction between DSS and time period ($p<0.001$), parent co-residence and related adults ($p<0.001$), parent co-residence and older siblings ($p<0.001$), and related adults and time period ($p=0.003$) significantly improved model fit. A multi-level model including a random intercept for the mother improved model fit according to the BIC ($\Delta BIC = 152$) and resulted in the final model.

Figure 5 shows the model-based predicted probabilities of a child dying by kin presence, parent co-residence, and time period. Having older siblings lowered the probability of dying only for children in a household with both parents (RRR=0.736 95% CI [0.633, 0.855]). While rare, having other adult kin present in two parent households resulted in a higher probability of dying for children. Only in the later ART period was there evidence that older adult kin lowered the probability of dying in single parent households (RRR=0.753 95% CI [0.664, 0.853]).

Sensitivity analysis

In the sub-models including household SES and household head gender, the main findings of household structure and composition remained (see online supplemental tables 3 and 4). Children in the highest (wealthiest) SES tertile had a lower risk of dying compared to children in the lowest (poorest) tertile.

For peer review only - http://bmjopen.bmj.com/site/about/guidelines.xhtml

DISCUSSION

Over a 16-year period, we examined associations between household structure and the role of kin in the household and differential mortality risk for children. Our main finding was that children in nuclear households with both parents had the lowest risk of dying. The kin presence model supported this finding, showing that having both parents as members of the household provided a protective effect for children.

The role of both parents is important to consider in light of the local context at both sites. Labour migration is common in both populations, and remittances play a beneficial role supporting children remaining in rural areas.[30] The protective role of the father has been shown in another comparative study from these sites,[2] highlighting their importance as long as they remain a breadwinner for the household.[31] However, our indicator of father presence does not capture the detail of their role and support in the household, particularly for those fathers non-coresident in the household.[32, 33] Female migration is also increasing over time,[34] with resulting changes in the associations with child mortality.[31] Most children remain residents of their migrating parent's origin household.[35] Further, the availability of government pensions allows children of migrants to stay with grandmothers who have resources to support them.

We showed that associations with kin and child mortality were moderated by parental status. We found a protective effect of related adults in one parent households only in the period of widespread ART availability. The lack of a protective effect in the earlier HIV period likely reflects complex household dynamics given excess AIDS mortality in prime aged adults.[23, 25] For instance, one parent households during this time period were more likely to have experienced loss of a parent. Related adults may have also placed additional burden on the household in this configuration,[36] for instance if they were unwell themselves. With widespread ART availability, the likelihood of parental loss and unwell related adults would both be reduced, which may reflect these adults being able to now provide caretaking and other roles that support vulnerable children.[16, 37] Presence of related adults may also help by reducing resource strain when funds are directed to unwell household members and providing care for children when mothers become very ill.[2]

For peer review only - http://bmjopen.bmj.com/site/about/guidelines.xhtml

Our findings suggest heterogeneity in the configuration of households and their associations with child mortality between the two sites. Household diversity has changed over time, in part due to low marriage rates, high levels of labour migration and unemployment, and high HIV prevalence.[38, 39] Results from reviews on the role of kin on child mortality have shown between study heterogeneity in kin effects, due in part to different study designs and lack of consistent controls.[40, 41] A strength of our study is the use of harmonized data and a unified statistical framework to examine differences in kin associations between the two sites. Our results provide evidence for the supportive role of kin, dependent on different ecological and epidemiological conditions that vary over time and between local contexts.

We acknowledge study limitations. First, our household variables only include matrilineal kin given the greater consistency over time of maternal identifiers. To create a harmonized data structure across the two sites, we also only identified household structure and composition based on memberships information. We therefore lack detailed information on the roles of matrilineal kin on child caretaking and nutrition, and how this may vary by parental status. Formative research is needed to understand the role of other household kin, particularly in one-parent households. Using household memberships also means that we cannot account for the role of kin who may provide child caretaking or support but are members of another household. Associations between household structure and composition may also be due to other unmeasured risk factors. We used multi-level modelling to account for shared mortality risk for children in the same household. Our results were also robust to controls for household SES and household head gender.

A key contribution of this study was to provide comparative, longitudinal evidence on associations between household structure and composition with child mortality from two rural South African populations both heavily burdened by HIV/AIDS. With the rollout of ART and rapid changes in SES,[23, 25, 29] these settings are continuing to undergo rapid demographic, epidemiological, and social transitions. Further longitudinal research is needed to understand continued changes in living arrangements and the role of parents and kin in protecting the well-being of children.

For peer review only - http://bmjopen.bmj.com/site/about/guidelines.xhtml

Ethics approval

Ethics approval for AHDSS was obtained from the Human Research Ethics Committee (Medical) of the University of the Witwatersrand, Johannesburg, South Africa (protocols M960720 and M110138). Ethics approval for AHRI was obtained from the Biomedical Research Ethics Committee (BREC) of the University of KwaZulu-Natal, Durban, South Africa (reference number BE169/15). Both sites obtain and document informed verbal consent at each census visit from the head of household or proxy adult respondent. This verbal consent process is standard across the INDEPTH Network of DSSs given the infeasibility of contacting every person in the DSS. The above ethics committees have continued to approve the verbal consenting process.

Data availability statement

The data underlying this article will be shared on reasonable request to the corresponding author. The data underlying the results presented in the study are available from the AHRI Data Repository (https://data.africacentre.ac.za) for researchers who meet the criteria for access to confidential data and sign on the agreement according to the AHRI's policy for data sharing. Detailed documentation of the AHDSS data and an anonymized database containing data from 10% of the surveillance households are available for public access on the AHDSS website (http://www.agincourt.za). The AHDSS core demographic data are also routinely deposited for public access in the INDEPTH Network Data Repository (http://www.indepthishare.org/) and the SAPRIN Data Repository (http://saprindata.samrc.ac.za/index.php/catalog). Customized data extraction can be requested from Dr. F. Xavier Gómez-Olivé (F.Gomez-OliveCasas@wits.ac.za).

Funding

The authors received no specific funding for this study. The MRC/Wits Rural Public Health and Health Transitions Research Unit and Agincourt Health and Socio-Demographic Surveillance System, a node of the South African Population Research Infrastructure Network (SAPRIN), is supported by the Department of Science and Innovation, the University of the Witwatersrand, and the Medical Research Council, South Africa, and previously the Wellcome Trust, UK (grants 058893/Z/99/A; 069683/Z/02/Z; 085477/Z/08/Z; 085477/B/08/Z). The Africa Health Research Institute is supported largely by Wellcome Trust (grant 201433/Z/16/Z) and Howard Hughes Medical Institute. The population surveillance, a node of the South African Population Research Infrastructure Network

For peer review only

(SAPRIN), is funded by the Department of Science and Innovation and hosted by the South African Medical Research Council.

Patient consent for publication

Not required.

Competing interests

None declared.

Contributors

BH and CWK conceived the study. BH wrote the first draft and designed and completed the statistical analyses. CWK prepared the data with the support of DG. KH and SJC provided overall guidance to the conduct of the study. CWK, DG, KH, and SJC revised the manuscript for important intellectual content and contributed to interpretation of the data. All authors read and approved the final manuscript.

For peer review only

REFERENCES

1. UN Inter-Agency Group for Child Mortality Estimation. Levels and Trends in Child Mortality: Report 2019. 2019. Available from: https://www.unicef.org/sites/default/files/2019-10/UN-IGME-child-mortality-report-2019.pdf
2. Houle B, Kabudula C, Stein A, Gareta D, Herst K, Clark S. Linking the timing of a mother's and child's death: Comparative evidence from two rural South African population-based surveillance studies, 2000-2015. *PLoS One* 2021;16:e0246671.
3. Tlou B, Sartorius B, Tanser F. Investigating risk factors for under-five mortality in an HIV hyper-endemic area of rural South Africa, from 2000-2014. *PLOS ONE* 2018;13:e0207294.
4. Zaba B, Whitworth J, Marston M et al. HIV and Mortality of Mothers and Children. *Epidemiology* 2005;16:275-280.
5. Ndirangu J, Newell ML, Tanser F, Herbst AJ, Bland R. Decline in early life mortality in a high HIV prevalence rural area of South Africa: evidence of HIV prevention or treatment impact? *AIDS* 2010;24:593-602.
6. Ndirangu J, Newell ML, Thorne C, Bland R. Treating HIV-infected mothers reduces under 5 years of age mortality rates to levels seen in children of HIV-uninfected mothers in rural South Africa. *Antivir Ther* 2012;17:81-90.
7. Newell M-L, Brahmbhatt H, Ghys PD. Child mortality and HIV infection in Africa: a review. *AIDS* 2004;18:S27-S34.
8. Desmond C, Michael K, Gow J. The Hidden Battle : HIV / AIDS in the Family and Community. *South African Journal of International Affairs* 2000;7:39-58.
9. Madhavan S, Townsend N. The social context of children's nutritional status in rural South Africa. *Scand J Public Health Suppl* 2007;69:107-117.
10. Madhavan S. Fosterage patterns in the age of AIDS: continuity and change. *Social science & medicine* 2004;58:1443-1454.
11. Schatz EJ. "Taking care of my own blood": older women's relationships to their households in rural South Africa. *Scand J Public Health Suppl* 2007;69:147-154.
12. Schatz E, Ogunmefun C. Caring and Contributing: The Role of Older Women in Rural South African Multi-generational Households in the HIV/AIDS Era. *World Development* 2007;35:1390-1403.
13. Gibson MA, Mace R. Helpful grandmothers in rural Ethiopia: A study of the effect of kin on child survival and growth. *Evolution and Human Behavior* 2005;26:469-482.
14. Omariba WR, Rajulton F, Beaujot R. Correlated mortality risks of siblings in Kenya. *Demographic Research* 2008;18:311-336.
15. Ronsmans C. Patterns of Clustering of Child Mortality in a Rural Area of Senegal. *Population Studies* 1995;49:443-461.
16. Sear R, Steele F, McGregor IA, Mace R. The effects of kin on child mortality in rural Gambia. *Demography* 2002;39:43-63.
17. van Dijk IK. Early-life mortality clustering in families: A literature review. *Popul Stud (Camb)* 20181-21.
18. Øyen N, Boyd HA, Poulsen G, Wohlfahrt J, Melbye M. The clustering of premature deaths in families. *Epidemiology* 2009;20:757-765.
19. Houle B, Stein A, Kahn K et al. Household context and child mortality in rural South Africa: the effects of birth spacing, shared mortality, household composition and socio-economic status. *Int J Epidemiol* 2013;42:1444-1454.

For peer review only - http://bmjopen.bmj.com/site/about/guidelines.xhtml

20. Horton R. The continuing invisibility of women and children. *The Lancet* 2010;375:1941-1943.
21. Madhavan S, Myroniuk TW, Kuhn R, Collinson MA. Household structure vs. composition: Understanding gendered effects on educational progress in rural South Africa. *Demogr Res* 2017;37:1891-1916.
22. Kahn K, Collinson MA, Gomez-Olive FX et al. Profile: Agincourt Health and Socio-demographic Surveillance System. *Int J Epidemiol* 2012;41:988-1001.
23. Kabudula C, Houle B, Ohene-Kwofie D et al. Mortality transition over a quarter century in rural South Africa: Finding from population surveillance in Agincourt 1993 — 2018. *Global Health Action* 2021;14:1990507.
24. Gareta D, Baisley K, M'ngomezulu T et al. Cohort profile update: Africa Centre Demographic Information System (ACDIS) and population-based HIV survey. *International Journal of Epidemiology* 202133-34f.
25. Reniers G, Blom S, Calvert C et al. Trends in the burden of HIV mortality after roll-out of antiretroviral therapy in KwaZulu-Natal, South Africa: an observational community cohort study. *The Lancet HIV* 2017;4:e113-e121.
26. Allison PD. Discrete-Time Methods for the Analysis of Event Histories. *Sociological Methodology* 1982;13:61-98.
27. Lumley T, Kronmal R, Ma S. Relative risk regression in medical research: Models, contrasts, estimators, and algorithms. *UW Biostatistics Working Paper Series* 2006;293
28. Byass P, Hussain-Alkhateeb L, D'Ambruoso L et al. An integrated approach to processing WHO-2016 verbal autopsy data: the InterVA-5 model. *BMC Medicine* 2019;17:102.
29. Kabudula C, Houle B, Collinson MA, Kahn K, Tollman S, Clark S. Assessing changes in household socioeconomic status in rural South Africa, 2001-2013: a distributional analysis using household asset indicators. *Social Indicators Research* 2017;133:1047-1073.
30. Collinson M. The Dynamics of Migration, Health and Livelihoods: INDEPTH Network Perspectives. 2009
31. Collinson MA. Striving against adversity: the dynamics of migration, health and poverty in rural South Africa. *Global Health Action* 2010;3:1-15.
32. Clark S, Cotton C, Marteleto LJ. Family Ties and Young Fathers' Engagement in Cape Town, South Africa. *Journal of Marriage and Family* 2015;77:575-589.
33. Madhavan S, Townsend NW, Garey AI. 'Absent Breadwinners': Father-Child Connections and Paternal Support in Rural South Africa. *J South Afr Stud* 2008;34:647-663.
34. Collinson MA, Tollman SM, Kahn K. Migration, settlement change and health in post-apartheid South Africa: Triangulating health and demographic surveillance with national census data1. *Scandinavian Journal of Public Health* 2007;35:77-84.
35. Bennett R, Hosegood V, Newell M, McGrath N. An approach to measuring dispersed families with a particular focus on children 'left behind'by migrant parents: findings from rural South Africa. *Population, space and place* 2015;21:322-334.
36. Basaza R, Kaija D, Ochola-Odonga D. The impact of HIV/AIDS on children: Lights and shadows in the "Successful Case" of Uganda. *AIDS, public policy and child well-being* 200731-66.
37. Cunningham SA, Elo IT, Herbst K, Hosegood V. Prenatal development in rural South Africa: relationship between birth weight and access to fathers and grandparents. *Popul Stud (Camb)* 2010;64:229-246.

For peer review only

38. Hosegood V. The demographic impact of HIV and AIDS across the family and household life-cycle: implications for efforts to strengthen families in sub-Saharan Africa. *AIDS Care* 2009;21 Suppl 1:13-21.
39. Madhavan S, Schatz EJ. Coping with change: household structure and composition in rural South Africa, 1992 - 2003. *Scand J Public Health Suppl* 2007;69:85-93.
40. Sear R, Mace R. Who keeps children alive? A review of the effects of kin on child survival. *Evolution and Human Behavior* 2008;29:1-18.
41. Strassmann BI, Garrard WM. Alternatives to the grandmother hypothesis: a meta-analysis of the association between grandparental and grandchild survival in patrilineal populations. *Hum Nat* 2011;22:201-222.

For peer review only

FIGURE CAPTIONS

Figure 1. Distribution of household structures over time and demographic surveillance site, Agincourt Health and Demographic Surveillance System (AHDSS) and Africa Health Research Institute (AHRI), South Africa 2000-2015.

Figure 2. Monthly probability of child death, by household structure and demographic surveillance site: Agincourt Health and Demographic Surveillance System (AHDSS) and Africa Health Research Institute (AHRI), South Africa 2000-2015. Jittered points to reduce over plotting.

Figure 3. Distribution of parent co-residence with children over time and demographic surveillance site, Agincourt Health and Demographic Surveillance System (AHDSS) and Africa Health Research Institute (AHRI), South Africa 2000-2015.

Figure 4. Proportion of children living with related adults and siblings (not mutually exclusive) over time and demographic surveillance site, Agincourt Health and Demographic Surveillance System (AHDSS) and Africa Health Research Institute (AHRI), South Africa 2000-2015.

Figure 5. Monthly probability of child death, by parent co-residence, kin presence, and time period: Agincourt Health and Demographic Surveillance System (AHDSS) and Africa Health Research Institute (AHRI), South Africa 2000-2015. Jittered points to reduce over plotting.

For peer review only

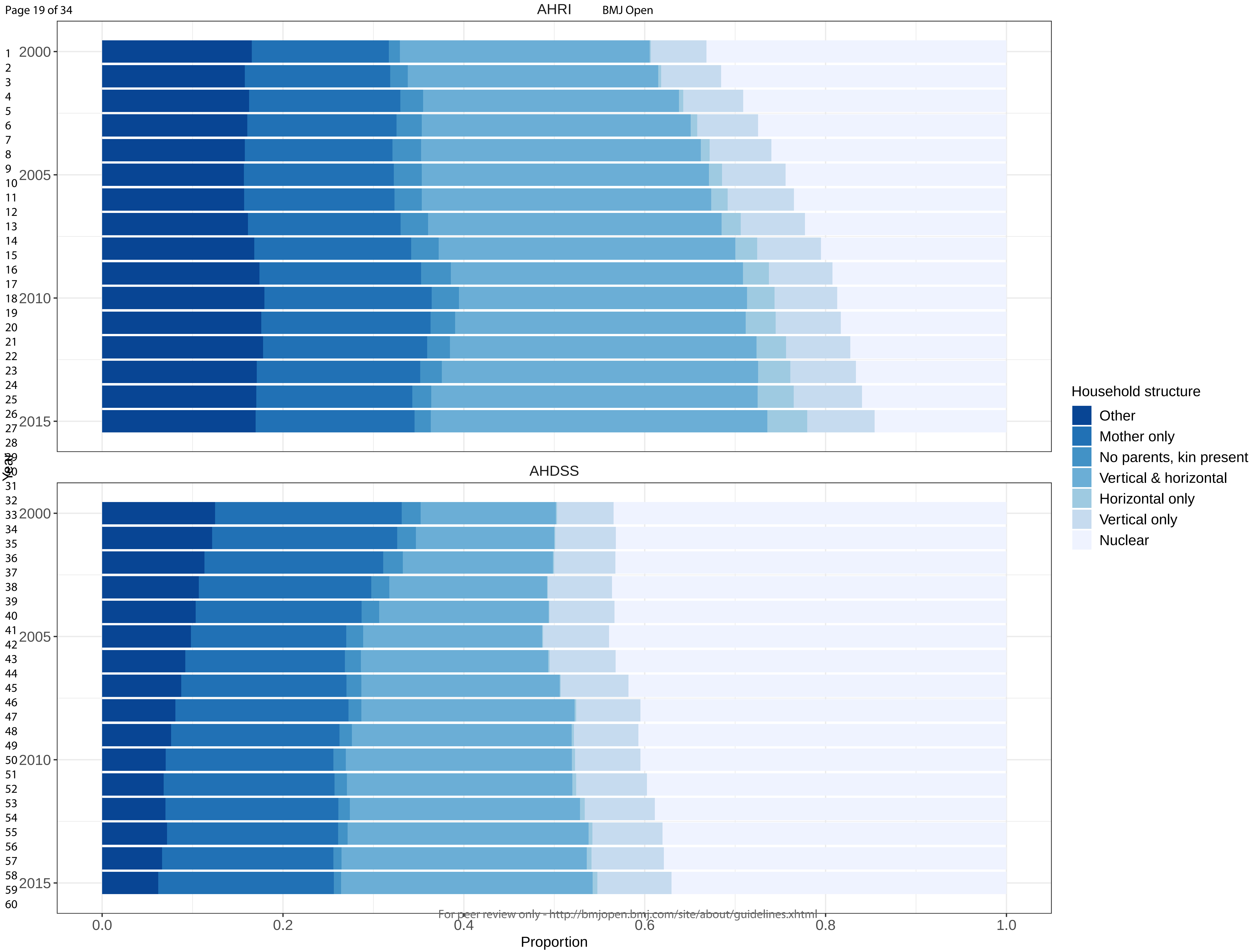
AHRI
AHDSS
Year
2000
2005
2010
2015
Proportion
0.0
0.2
0.4
0.6
0.8
1.0
Household structure
Other
Mother only
No parents, kin present
Vertical & horizontal
Horizontal only
Vertical only
Nuclear

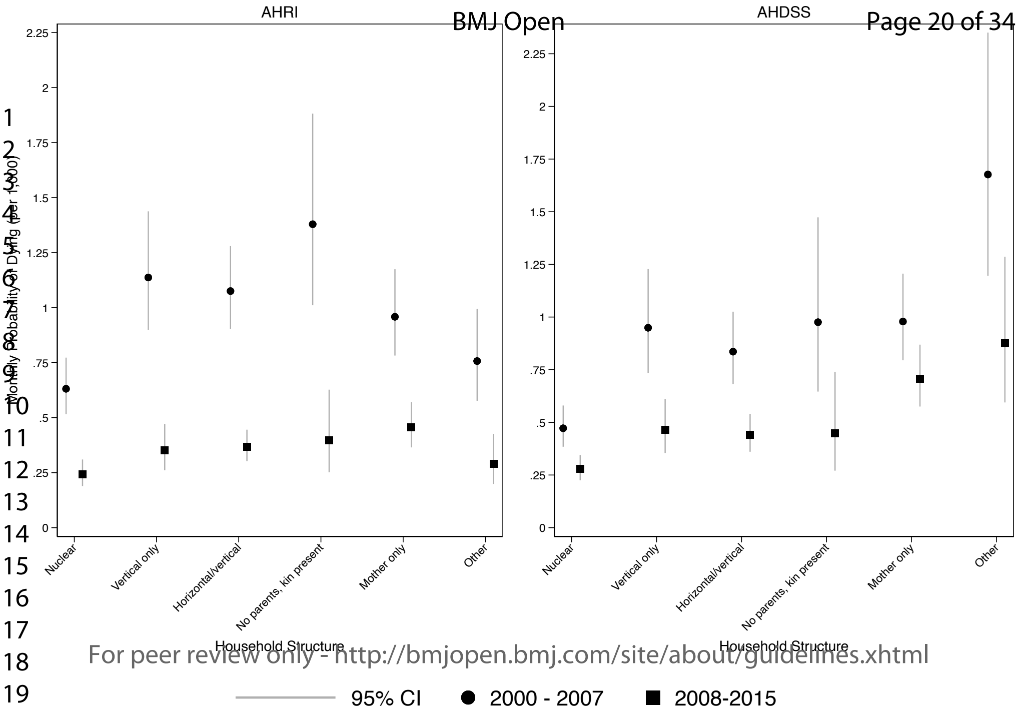
AHRI
AHDSS
Monthly Probability of Dying (per 1,000)
0
.25
.5
.75
1
1.25
1.5
1.75
2
2.25
Nuclear
Vertical only
Horizontal/vertical
No parents, kin present
Mother only
Other
Household Structure
95% CI
2000 - 2007
2008-2015

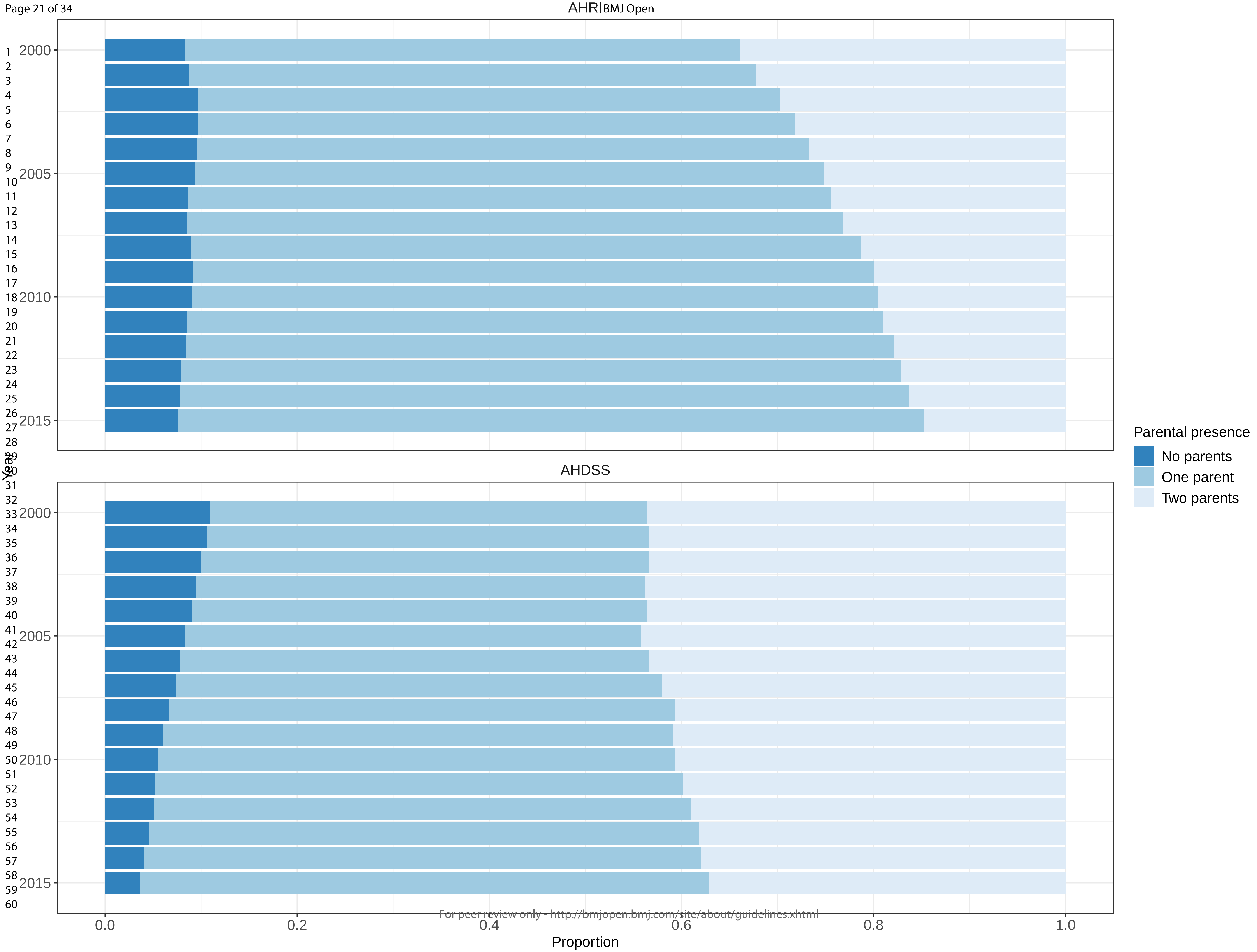

For peer review only - http://bmjopen.bmj.com/site/about/guidelines.xhtml

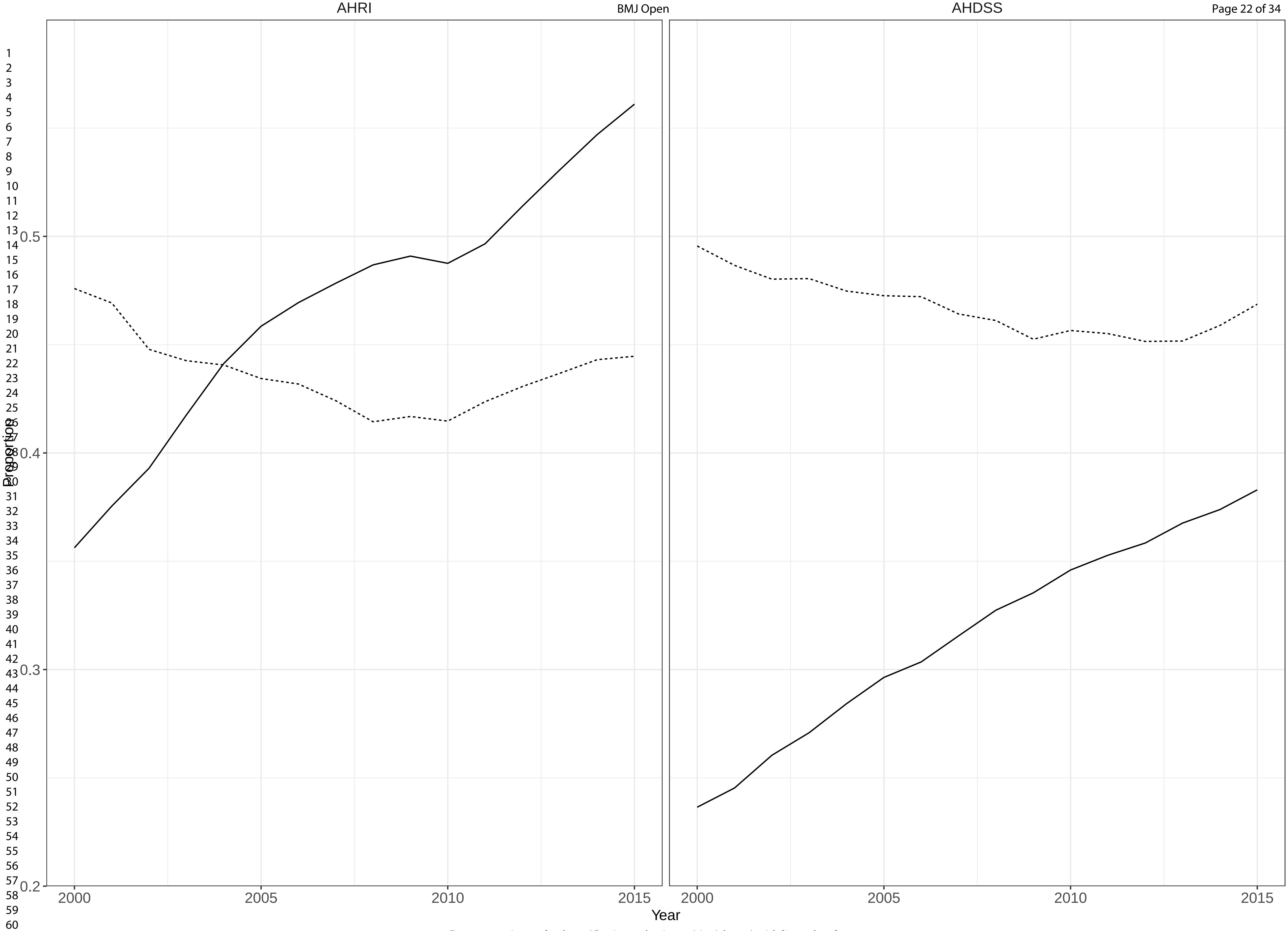
AHRI
AHDSS
Proportion
0.5
0.4
0.3
0.2
2000
2005
2010
2015
Year
Related adults
Siblings

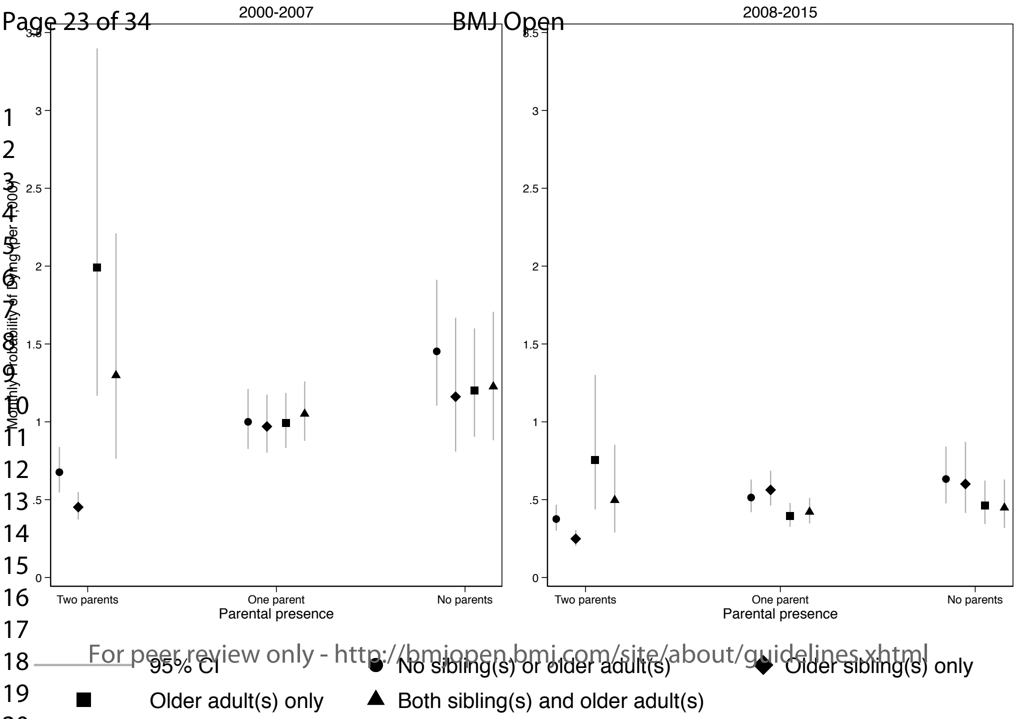
2000-2007
2008-2015
Monthly Probability of Dying (per 1,000)
0
.5
1
1.5
2
2.5
3
3.5
Two parents
One parent
No parents
Parental presence
95% CI
No sibling(s) or older adult(s)
Older sibling(s) only
Older adult(s) only
Both sibling(s) and older adult(s)

Supplemental Figure 1. Child mortality rates by year, Agincourt Health and Demographic Surveillance System (AHDSS) and Africa Health Research Institute (AHRI), South Africa 2000-2015.

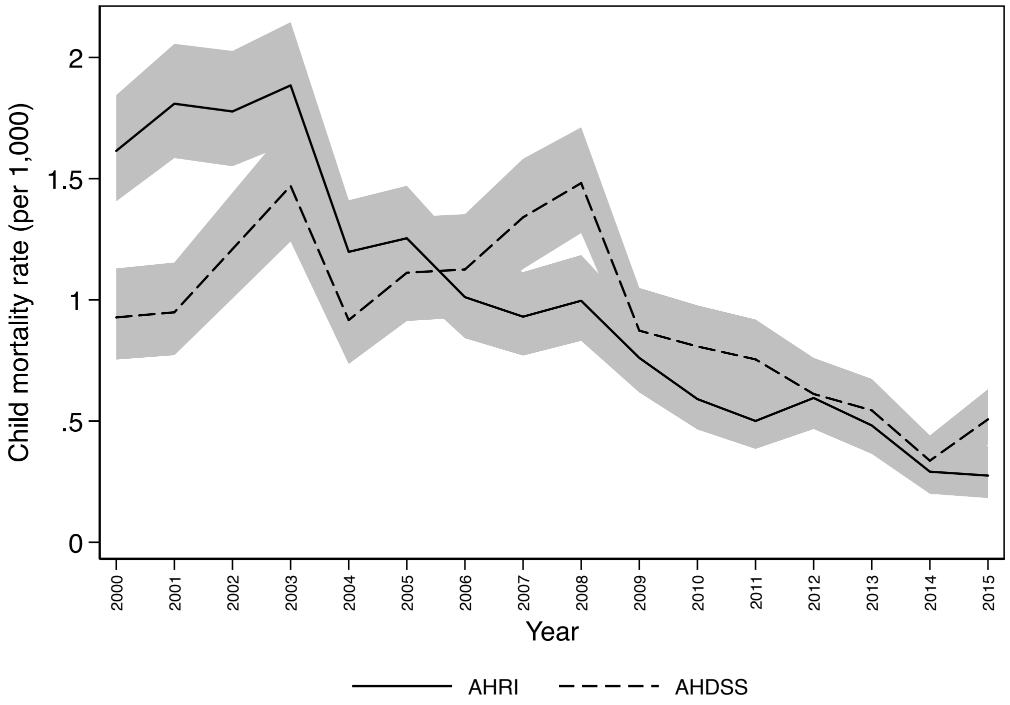

Supplemental Figure 2. Distribution of child causes of death over time, Agincourt Health and Demographic Surveillance System (AHDSS) and Africa Health Research Institute (AHRI), South Africa 2000-2007 and 2008-2015. Child causes of death classified by InterVA-5 based on VA.

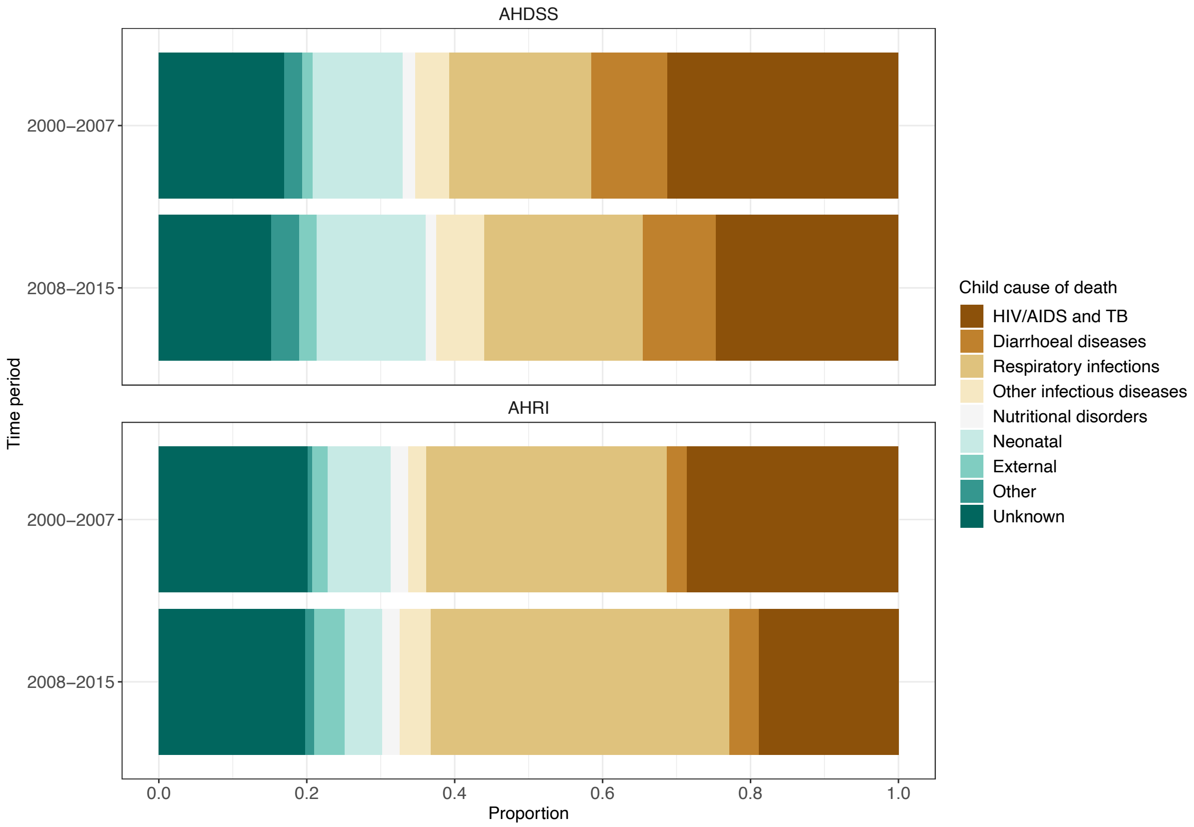

Supplemental Table 1. Multilevel relative risk regression of child death on household structure and controls, Agincourt Health and Demographic Surveillance System (AHDSS) and Africa Health Research Institute (AHRI), South Africa 2000-2015 (n=3,637,638 child months).

	RRR	95% CI	p-value
Site			
AHRI	1	-	
AHDSS	0.757	[0.647, 0.887]	0.001
Time period			
2000 to 2007	1	-	
2008 to 2015	0.415	[0.346, 0.498]	<0.001
Site X time period			
AHDSS X 2008 to 2015	1.454	[1.251, 1.689]	<0.001
Sex of child			
Female	1	-	
Male	1.124	[1.049, 1.204]	0.001
Child age (months)			
<1	1	-	
1 to 6	0.38	[0.342, 0.423]	<0.001
7 to 23	0.177	[0.159, 0.196]	<0.001
24 to 59	0.035	[0.031, 0.039]	<0.001
Multiple birth			
Singleton	1	-	
Multiple birth	1.818	[1.557, 2.124]	<0.001
Mother's age at birth (years			
15-19	0.864	[0.780, 0.956]	0.005
20-24	1	-	
25-29	1.264	[1.148, 1.392]	<0.001
30-34	1.089	[0.969, 1.225]	0.152
35+	1.237	[1.091, 1.403]	0.001
Number ages 0-5			
0	1	-	
1	1.098	[1.011, 1.192]	0.027
2+	1.251	[1.138, 1.376]	<0.001
Number ages 5-19			
0	1	-	
1 to 2	0.98	[0.849, 1.132]	0.787
3 to 4	0.928	[0.800, 1.076]	0.321
5+	1.044	[0.894, 1.219]	0.588
Number ages 20+			
0 to 2	1	-	
3 to 4	0.975	[0.879, 1.081]	0.631
5+	0.938	[0.841, 1.046]	0.249

For peer review only

Household structure			
Nuclear	1	-	
Vertical only	1.644	[1.325, 2.041]	<0.001
Horizontal/vertical	1.635	[1.408, 1.898]	<0.001
No parents, kin present	2.906	[2.169, 3.895]	<0.001
Mother only	1.554	[1.313, 1.839]	<0.001
Other	1.809	[1.411, 2.319]	<0.001
Site X household structure			
AHDSS X Vertical only	1.181	[0.892, 1.563]	0.245
AHDSS X Horizontal/vertical	1.07	[0.877, 1.305]	0.506
AHDSS X No parents, kin present	0.938	[0.595, 1.479]	0.784
AHDSS X Mother only	1.373	[1.112, 1.696]	0.003
AHDSS X Other	1.932	[1.339, 2.787]	<0.001
Time period X household structure			
2008 to 2015 X Vertical only	0.842	[0.632, 1.122]	0.241
2008 to 2015 X Horizontal/vertical	0.905	[0.742, 1.104]	0.324
2008 to 2015 X No parents, kin present	0.818	[0.506, 1.323]	0.413
2008 to 2015 X Mother only	1.2	[0.973, 1.479]	0.089
2008 to 2015 X Other	1.07	[0.725, 1.578]	0.734
	Parameter		
σ^2_{mother}	3.152	[2.574, 3.859]	

For peer review only

Supplemental Table 2. Multilevel relative risk regression of child death on kin presence and controls, Agincourt Health and Demographic Surveillance System (AHDSS) and Africa Health Research Institute (AHRI), South Africa 2000-2015 (n=3,637,638 child months).

	RRR	95% CI	p-value
Site			
AHRI	1	-	
AHDSS	0.874	[0.794, 0.961]	0.006
Time period			
2000 to 2007	1	-	
2008 to 2015	0.456	[0.399, 0.520]	<0.001
Site X time period			
AHDSS X 2008 to 2015	1.437	[1.241, 1.664]	<0.001
Sex of child			
Female	1	-	
Male	1.125	[1.050, 1.205]	0.001
Child age (months)			
<1	1	-	
1 to 6	0.379	[0.341, 0.422]	<0.001
7 to 23	0.176	[0.159, 0.195]	<0.001
24 to 59	0.035	[0.031, 0.039]	<0.001
Multiple birth			
Singleton	1	-	
Multiple birth	1.804	[1.545, 2.106]	<0.001
Mother's age at birth (years			
15-19	0.866	[0.780, 0.961]	0.007
20-24	1	-	
25-29	1.277	[1.155, 1.413]	<0.001
30-34	1.127	[0.995, 1.278]	0.06
35+	1.309	[1.143, 1.499]	<0.001
Number ages 0-5			
0	1	-	
1	1.096	[1.009, 1.190]	0.029
2+	1.249	[1.136, 1.373]	<0.001
Number ages 5-19			
0	1	-	
1 to 2	1.069	[0.917, 1.246]	0.395
3 to 4	1.014	[0.864, 1.189]	0.865
5+	1.147	[0.969, 1.357]	0.11
Number ages 20+			
0 to 2	1	-	
3 to 4	0.953	[0.861, 1.056]	0.359
5+	0.903	[0.811, 1.004]	0.06
Parental presence			

For peer review only

Both parents	1	-	
One parent	1.569	[1.363, 1.807]	<0.001
Neither parent	2.623	[2.058, 3.343]	<0.001
Related adult presence			
No	1	-	
Yes	2.954	[1.769, 4.931]	<0.001
Parental presence X related adult presence			
One parent X related adult present	0.316	[0.188, 0.530]	<0.001
Neither parent X related adult present	0.307	[0.171, 0.551]	<0.001
Related siblings			
No	1	-	
Yes	0.712	[0.606, 0.837]	<0.001
Parental presence X related siblings			
One parent X related siblings present	1.464	[1.233, 1.737]	<0.001
Neither parent X related siblings present	1.518	[1.070, 2.153]	0.019
Time period X related adult presence			
2008 to 2015 X related adult presence	0.806	[0.698, 0.932]	0.004
	Parameter		
σ^2_{mother}	1.128	[0.944, 1.349]	

For peer review only

Supplemental Table 3. Multilevel relative risk regression of child death on household structure and controls, Agincourt Health and Demographic Surveillance System (AHDSS) and Africa Health Research Institute (AHRI), South Africa 2000-2015 (n=3,637,638 child months). Estimation sample restricted to those with household SES information.

	**RRR**	**95% CI**	**p-value**
Site			
AHRI	1	-	
AHDSS	0.772	[0.625, 0.954]	0.017
Time period			
2000 to 2007	1	-	
2008 to 2015	0.391	[0.305, 0.501]	<0.001
Site X time period			
AHDSS X 2008 to 2015	1.395	[1.141, 1.705]	0.001
Sex of child			
Female	1	-	
Male	1.134	[1.037, 1.240]	0.006
Child age (months)			
<1	1	-	
1 to 6	0.355	[0.304, 0.415]	<0.001
7 to 23	0.176	[0.152, 0.205]	<0.001
24 to 59	0.036	[0.030, 0.042]	<0.001
Multiple birth			
Singleton	1	-	
Multiple birth	1.58	[1.281, 1.948]	<0.001
Mother's age at birth (years			
15-19	0.897	[0.785, 1.024]	0.107
20-24	1	-	
25-29	1.364	[1.202, 1.548]	<0.001
30-34	1.175	[1.008, 1.368]	0.039
35+	1.391	[1.183, 1.634]	<0.001
Number ages 0-5			
0	1	-	
1	1.049	[0.943, 1.167]	0.375
2+	1.225	[1.082, 1.387]	0.001
Number ages 5-19			
0	1	-	
1 to 2	1.031	[0.855, 1.243]	0.746
3 to 4	0.949	[0.782, 1.152]	0.594
5+	1.034	[0.843, 1.268]	0.748
Number ages 20+			
0 to 2	1	-	
3 to 4	0.912	[0.798, 1.041]	0.174
5+	0.868	[0.753, 1.002]	0.053

For peer review only

Household structure			
Nuclear	1	-	
Vertical only	1.851	[1.374, 2.495]	<0.001
Horizontal/vertical	1.733	[1.408, 2.131]	<0.001
No parents, kin present	3.356	[2.292, 4.914]	<0.001
Mother only	1.716	[1.357, 2.170]	<0.001
Other	1.892	[1.335, 2.680]	<0.001
Site X household structure			
AHDSS X Vertical only	1.099	[0.752, 1.604]	0.627
AHDSS X Horizontal/vertical	1.127	[0.863, 1.470]	0.38
AHDSS X No parents, kin present	1.072	[0.619, 1.857]	0.804
AHDSS X Mother only	1.309	[0.981, 1.746]	0.067
AHDSS X Other	2.069	[1.274, 3.360]	0.003
Time period X household structure			
2008 to 2015 X Vertical only	0.734	[0.502, 1.075]	0.112
2008 to 2015 X Horizontal/vertical	0.838	[0.648, 1.085]	0.181
2008 to 2015 X No parents, kin present	0.763	[0.416, 1.399]	0.382
2008 to 2015 X Mother only	1.278	[0.978, 1.669]	0.073
2008 to 2015 X Other	1.044	[0.624, 1.745]	0.87
Household SES			
Low	1	-	
Middle	0.949	[0.852, 1.058]	0.346
High	0.788	[0.702, 0.883]	<0.001
Household head gender			
Female	1	-	
Male	1.104	[0.999, 1.220]	0.052
	Parameter		
σ^2_{mother}	3.152	[2.574, 3.859]	

For peer review only

Supplemental Table 4. Multilevel relative risk regression of child death on kin presence and controls, Agincourt Health and Demographic Surveillance System (AHDSS) and Africa Health Research Institute (AHRI), South Africa 2000-2015 (n=2,468,466 child months). Estimation sample restricted to those with household SES information.

	**RRR**	**95% CI**	**p-value**
Site			
AHRI	1	-	
AHDSS	0.903	[0.797, 1.022]	0.106
Time period			
2000 to 2007	1	-	
2008 to 2015	0.442	[0.367, 0.532]	<0.001
Site X time period			
AHDSS X 2008 to 2015	1.374	[1.129, 1.672]	0.002
Sex of child			
Female	1	-	
Male	1.136	[1.039, 1.242]	0.005
Child age (months)			
<1	1	-	
1 to 6	0.354	[0.303, 0.414]	<0.001
7 to 23	0.176	[0.152, 0.204]	<0.001
24 to 59	0.036	[0.030, 0.042]	<0.001
Multiple birth			
Singleton	1	-	
Multiple birth	1.571	[1.275, 1.936]	<0.001
Mother's age at birth (years			
15-19	0.889	[0.775, 1.019]	0.091
20-24	1	-	
25-29	1.4	[1.227, 1.597]	<0.001
30-34	1.246	[1.059, 1.466]	0.008
35+	1.523	[1.280, 1.812]	<0.001
Number ages 0-5			
0	1	-	
1	1.046	[0.941, 1.163]	0.406
2+	1.219	[1.077, 1.380]	0.002
Number ages 5-19			
0	1	-	
1 to 2	1.19	[0.974, 1.454]	0.089
3 to 4	1.103	[0.894, 1.360]	0.36
5+	1.212	[0.971, 1.513]	0.089
Number ages 20+			
0 to 2	1	-	
3 to 4	0.89	[0.780, 1.015]	0.081
5+	0.827	[0.719, 0.951]	0.008

For peer review only

Parental presence			
Both parents	1	-	
One parent	1.609	[1.341, 1.930]	<0.001
Neither parent	2.865	[2.086, 3.933]	<0.001
Related adult presence			
No	1	-	
Yes	4.031	[2.137, 7.604]	<0.001
Parental presence X related adult presence			
One parent X related adult present	0.228	[0.120, 0.432]	<0.001
Neither parent X related adult present	0.229	[0.110, 0.474]	<0.001
Related siblings			
No	1	-	
Yes	0.6	[0.487, 0.739]	<0.001
Parental presence X related siblings			
One parent X related siblings present	1.695	[1.360, 2.113]	<0.001
Neither parent X related siblings present	1.84	[1.185, 2.856]	0.007
Time period X related adult presence			
2008 to 2015 X related adult presence	0.722	[0.599, 0.871]	0.001
Household SES			
Low	1	-	
Middle	0.946	[0.849, 1.054]	0.314
High	0.784	[0.699, 0.879]	<0.001
Household head gender			
Female	1	-	
Male	1.107	[1.004, 1.221]	0.042
	Parameter		
σ^2_{mother}	3.375	[2.494, 4.568]	

For peer review only

STROBE Statement—checklist of items that should be included in reports of observational studies

	Item No	Recommendation	Page No
**Title and abstract**	1	(*a*) Indicate the study's design with a commonly used term in the title or the abstract	1
		(*b*) Provide in the abstract an informative and balanced summary of what was done and what was found	2
**Introduction**			
Background/rationale	2	Explain the scientific background and rationale for the investigation being reported	4
Objectives	3	State specific objectives, including any prespecified hypotheses	4-5
**Methods**			
Study design	4	Present key elements of study design early in the paper	5
Setting	5	Describe the setting, locations, and relevant dates, including periods of recruitment, exposure, follow-up, and data collection	5
Participants	6	(*a*) *Cohort study*—Give the eligibility criteria, and the sources and methods of selection of participants. Describe methods of follow-up<br>*Case-control study*—Give the eligibility criteria, and the sources and methods of case ascertainment and control selection. Give the rationale for the choice of cases and controls<br>*Cross-sectional study*—Give the eligibility criteria, and the sources and methods of selection of participants	6
		(*b*) *Cohort study*—For matched studies, give matching criteria and number of exposed and unexposed<br>*Case-control study*—For matched studies, give matching criteria and the number of controls per case	
Variables	7	Clearly define all outcomes, exposures, predictors, potential confounders, and effect modifiers. Give diagnostic criteria, if applicable	6
Data sources/ measurement	8*	For each variable of interest, give sources of data and details of methods of assessment (measurement). Describe comparability of assessment methods if there is more than one group	5-7
Bias	9	Describe any efforts to address potential sources of bias	7
Study size	10	Explain how the study size was arrived at	6-7
Quantitative variables	11	Explain how quantitative variables were handled in the analyses. If applicable, describe which groupings were chosen and why	6-7
Statistical methods	12	(*a*) Describe all statistical methods, including those used to control for confounding	6-7
		(*b*) Describe any methods used to examine subgroups and interactions	6-7
		(*c*) Explain how missing data were addressed	6
		(*d*) *Cohort study*—If applicable, explain how loss to follow-up was addressed<br>*Case-control study*—If applicable, explain how matching of cases and controls was addressed<br>*Cross-sectional study*—If applicable, describe analytical methods taking account of sampling strategy	
		(*e*) Describe any sensitivity analyses	7

Continued on next page

For peer review only - http://bmjopen.bmj.com/site/about/guidelines.xhtml

**Results**			
Participants	13*	(a) Report numbers of individuals at each stage of study—eg numbers potentially eligible, examined for eligibility, confirmed eligible, included in the study, completing follow-up, and analysed	
		(b) Give reasons for non-participation at each stage	
		(c) Consider use of a flow diagram	
Descriptive data	14*	(a) Give characteristics of study participants (eg demographic, clinical, social) and information on exposures and potential confounders	Fig1,3,4
		(b) Indicate number of participants with missing data for each variable of interest	
		(c) *Cohort study*—Summarise follow-up time (eg, average and total amount)	
Outcome data	15*	*Cohort study*—Report numbers of outcome events or summary measures over time	
		Case-control study—Report numbers in each exposure category, or summary measures of exposure	
		Cross-sectional study—Report numbers of outcome events or summary measures	
Main results	16	(*a*) Give unadjusted estimates and, if applicable, confounder-adjusted estimates and their precision (eg, 95% confidence interval). Make clear which confounders were adjusted for and why they were included	
		(*b*) Report category boundaries when continuous variables were categorized	
		(*c*) If relevant, consider translating estimates of relative risk into absolute risk for a meaningful time period	
Other analyses	17	Report other analyses done—eg analyses of subgroups and interactions, and sensitivity analyses	9
**Discussion**			
Key results	18	Summarise key results with reference to study objectives	9
Limitations	19	Discuss limitations of the study, taking into account sources of potential bias or imprecision. Discuss both direction and magnitude of any potential bias	10-11
Interpretation	20	Give a cautious overall interpretation of results considering objectives, limitations, multiplicity of analyses, results from similar studies, and other relevant evidence	11
Generalisability	21	Discuss the generalisability (external validity) of the study results	10
**Other information**			
Funding	22	Give the source of funding and the role of the funders for the present study and, if applicable, for the original study on which the present article is based	12

*Give information separately for cases and controls in case-control studies and, if applicable, for exposed and unexposed groups in cohort and cross-sectional studies.

Note: An Explanation and Elaboration article discusses each checklist item and gives methodological background and published examples of transparent reporting. The STROBE checklist is best used in conjunction with this article (freely available on the Web sites of PLoS Medicine at http://www.plosmedicine.org/, Annals of Internal Medicine at http://www.annals.org/, and Epidemiology at http://www.epidem.com/). Information on the STROBE Initiative is available at www.strobe-statement.org.

For peer review only
